# Field detection devices for screening the quality of medicines: a systematic review

**DOI:** 10.1136/bmjgh-2018-000725

**Published:** 2018-08-29

**Authors:** Serena Vickers, Matthew Bernier, Stephen Zambrzycki, Facundo M Fernandez, Paul N Newton, Céline Caillet

**Affiliations:** 1 Lao-Oxford-Mahosot Hospital-Wellcome Trust Research Unit (LOMWRU), Microbiology Laboratory, Mahosot Hospital, Vientiane, Laos; 2 Centre for Tropical Medicine and Global Health, University of Oxford, Oxford, UK; 3 Infectious Diseases Data Observatory (IDDO)/Worldwide Antimalarial Resistance Network (WWARN), University of Oxford, Oxford, UK; 4 School of Chemistry and Biochemistry, Georgia Institute of Technology, Atlanta, Georgia, USA; 5 Campus Chemical Instrument Center Mass Spectrometry and Proteomics Facility, The Ohio State University, Columbus, Ohio, USA

**Keywords:** other diagnostic or tool, control strategies, public health, screening, systematic review

## Abstract

**Background:**

Poor quality medicines have devastating consequences. A plethora of innovative portable devices to screen for poor quality medicines has become available, leading to hope that they could empower medicine inspectors and enhance surveillance. However, information comparing these new technologies is woefully scarce.

**Methods:**

We undertook a systematic review of Embase, PubMed, Web of Science and SciFinder databases up to 30 April 2018. Scientific studies evaluating the performances/abilities of portable devices to assess any aspect of the quality of pharmaceutical products were included.

**Results:**

Forty-one devices, from small benchtop spectrometers to ‘lab-on-a-chip’ single-use devices, with prices ranging from <US$10 to >US$20 000, were included. Only six devices had been field-tested (GPHF-Minilab, CD3/CD3+, TruScan RM, lateral flow dipstick immunoassay, CBEx and Speedy Breedy). The median (range) number of active pharmaceutical ingredients (APIs) assessed per device was only 2 (1–20). The majority of devices showed promise to distinguish genuine from falsified medicines. Devices with the potential to assay API (semi)-quantitatively required consumables and were destructive (GPHF-Minilab, PharmaChk, aPADs, lateral flow immunoassay dipsticks, paper-based microfluidic strip and capillary electrophoresis), except for spectroscopic devices. However, the 10 spectroscopic devices tested for their abilities to quantitate APIs required processing complex API-specific calibration models. Scientific evidence of the ability of the devices to accurately test liquid, capsule or topical formulations, or to distinguish between chiral molecules, was limited. There was no comment on cost-effectiveness and little information on where in the pharmaceutical supply chain these devices could be best deployed.

**Conclusion:**

Although a diverse range of portable field detection devices for medicines quality screening is available, there is a vitally important lack of independent evaluation of the majority of devices, particularly in field settings. Intensive research is needed in order to inform national medicines regulatory authorities of the optimal choice of device(s) to combat poor quality medicines.

**Key questions**

**What is already known?**

- ~10% of medical products circulating in low-income and middle-income countries are either substandard or falsified, leading to increased morbidity and mortality, adverse drug reactions, economic losses and diminished public confidence in health systems.
- A large number of portable screening devices have recently been developed that could aid medicines regulatory authorities in the detection of poor quality medicines, but there is scanty evidence to inform policy makers as to which device to use and where.

**What are the new findings?**

- Forty-one devices covering 19 technologies were identified; more than half of these devices employed spectroscopic techniques.
- Field evaluation has been published for only 6 of 41 devices and
- ignificant knowledge gaps exist, impairing evidence-based policy decisions.

**What do the new findings imply?**

- There is inadequate independent evaluation of these devices to inform policy makers about optimal choice of device to combat poor quality medicines.
- Intensive research is needed to understand the comparative advantages and limitations of the different devices and technologies.

## Introduction

According to a recent WHO report, ~10% of medical products circulating in low-income and middle-income countries (L/MICs) are either substandard or falsified.^1^ Although this problem is as old as the medicinal trade,^2 3^ its impact on global health has been largely under-recognised. L/MICs are significantly affected,^4–6^ but wealthier countries with good regulatory systems are not immune.^7–9^ Substandard and falsified (SF) medicines (box 1) have devastating consequences, including increased morbidity and mortality, economic losses and diminished public confidence in health systems. Poor quality antimicrobials, particularly those containing reduced quantities of active pharmaceutical ingredients (APIs), may be a key but neglected driver of antimicrobial resistance (AMR).^10^ Despite this, the oversight and penalties for perpetrators are weak, and falsifying medicines remains an attractive criminal activity.^11^

Box 1 **Definitions of substandard and falsified medicines^86^**

- *Substandard*, also called ‘out of specification’: authorised medical products that fail to meet either their quality standards or specifications, or both.
- *Falsified*: medical products that deliberately/fraudulently misrepresent their identity, composition or source.

Medicines regulatory authorities (MRAs) are responsible for preventing, detecting and removing SF medicines. Others actors involved in medicine procurement (eg, non-governmental organisations, procurement agencies and hospital pharmacies) are, together with MRAs, keystones for the majority of potential interventions to prevent, detect and remove poor quality medicines. Currently, in L/MICs these key actors often have only their own senses and knowledge to rely on as they seek circulating SF medicines. Samples may then be sent for formal chemical analysis laboratory testing, using API-specific (and dosage formulation-specific) validated pharmacopeial protocols, or non-validated inhouse procedures when pharmacopeial methods do not exist. However, these tests (such as high-performance liquid chromatography (HPLC)) are expensive, time-consuming and scarce in many countries (box 2). There are often significant delays between collection of suspicious medicines and confirmation of their poor quality, with harm spreading unchecked in the interim.

Box 2 **Main technologies used in pharmaceutical quality analysis**

**Colourimetry**

- Colourimetric techniques use analysis of the colour developed by a sample in the presence of specific reagents. The presence or absence of the colour gives information on the presence or absence of the chemical compound (or specific chemical groups) being investigated. The intensity of the colour, interpreted either by the naked eye or by specific devices (called colourimeters or photometers), can provide quantitative information on the amount of the chemical within the medicine.

**Chromatography**

- This technology separates different ingredients in a mixture to obtain pure compounds to show their presence (or absence) and quantity. As many compounds are colourless, specific detectors are used to reveal them, such as those based on refraction index changes, fluorescence or absorbance at various wavelengths.
- Individual compounds are separated from each other through their interaction with a solid ‘stationary phase’, which remains fixed in a column or support. A liquid or a gas ‘mobile phase’ flows through the stationary phase and the captured compounds gradually move along the stationary phase in the same direction as the mobile phase. Each compound of a mixture will travel through the stationary phase, ejecting at varying times due to their different affinities with the stationary versus mobile phases. For examining medicines quality, the result from the test sample is compared with the result yielded by the authentic product, tested under the same conditions.
- *Thin-layer chromatography* uses a thin layer of silica or paper as the stationary phase. The mobile phase travels through the stationary phase via capillary action when the base of the device is placed with one end dipped in a solution. Once the device is pulled from the solution, the separation process stops and the separated compounds are retained spatially on the stationary phase, and revealed with the use of a lamp or chemical reagent.
- *High-performance liquid chromatography* forces the mobile phase through a column of stationary phase silica particles by high-pressure pumps. A detector monitors the compounds as they are released, allowing the identification of the compounds based on their specific retention times and quantitation based on their peak area. Typical detectors vary in terms of cost and specificity, and include UV-Vis light absorbance detectors and (quadrupole, ion trap, time-of-flight, orbitrap) mass spectrometers.

**Spectroscopy: near-infrared (NIR), mid-infrared (MIR), Raman and ultraviolet-visible light (UV-Vis)**

- Different chemicals have their own unique interaction with electromagnetic radiation. The type of interaction depends on the nature of the compound’s molecular structure and the radiation used. When a sample is irradiated with a specific wavelength (energy) of light, structures within the sample absorb that energy and vibrate along different chemical bonds which can be measured by *NIR*, *MIR* and *Raman spectroscopy*, types of ‘*vibrational spectroscopy*’. These ‘vibrations’ cause the absorbance or emittance of light by the sample in a characteristic spectrum, unique to the sample—often called a ‘spectral fingerprint’. Usually, this unique spectrum has to undergo mathematical transformation (spectral processing) to be readable by the user. In order to identify whether a sample is authentic or substandard/falsified, the sample spectrum generated is compared with the spectrum of the authentic product to assess its similarity. This requires the construction of a ‘reference library’ or database consisting of the spectra of authentic products.
- *UV-Vis* spectroscopy uses light within the ultraviolet and visible regions of the electromagnetic spectrum. UV-Vis absorbance measurements, which monitor the amount of light within this part of the spectrum that is transmitted through a material, do not reveal as much structural information as *NIR*, *MIR* and *Raman*. However, fluorescence and luminescence signals can be measured within the *UV-Vis* region. Signals from samples that can emit *UV-Vis* light through fluorescence (ie, the sample can be excited by a wavelength of light and then emit a different wavelength of light) and luminescence (a chemical reaction emits light from the sample) can thus be used to characterise and quantify the amount of active pharmaceutical ingredients within a sample.

**Structurally based separation techniques**

- Molecules of different mass and charge move differently when under the influence of an external electric field (heavier molecules travel slower or require stronger electric fields to be transported). These travel times or electric field conditions are recorded by a detector and are correlated to the mass and charge of the molecule, allowing its identification. *Mass spectrometry*, *ion mobility spectroscopy* and *capillary electrophoresis* all exploit this phenomenon. Mass spectrometry measures movement through a vacuum, ion mobility in the gas phase and capillary electrophoresis in the liquid phase. For examining medicines quality, the result from the test sample is compared with the result yielded by the authentic product, tested under the same conditions.

Over the last two decades a plethora of portable medicine analysis screening tools have been developed, offering the potential for objective analysis of medicines in the ‘field’. A previous review compared the suitability of different existing chemical analysis technologies for L/MICs^12^ (eg, Raman spectroscopy, colourimetry). With more devices and more data now available, we undertook a systematic review to understand the performance and characteristics of portable devices for the field evaluation of medicines. This review identifies multiple gaps in the evidence for optimal device selection to inform policy decisions on which devices to use to screen medicine quality before sending samples for confirmatory analyses, where and when.

## Methods

### Search strategy and selection criteria

A systematic review was conducted, following the Preferred Reporting Items for Systematic Reviews and Meta-Analyses guidelines (online supplementary file 1, PRISMA checklist) with registration in the international prospective register of systematic reviews (PROSPERO, ID 42016043216). We searched for English-language scientific articles on portable technologies used to assess the quality of pharmaceutical products, using Embase (from 1947), PubMed (from 1946), Web of Science (from 1900) and SciFinder (from 1840) to 30 April 2018. Search terms included those related to the equipment (eg, ‘device’, ‘instrument’), terms referring to the portability of the equipment (eg, ‘portable’, ‘handheld’) and terms related to the quality of pharmaceutical products (eg, ‘substandard’, ‘falsified’). The full search strategies are provided in online supplementary file 2.

10.1136/bmjgh-2018-000725.supp1 Supplementary data

10.1136/bmjgh-2018-000725.supp2 Supplementary data

After removal of duplicates, titles and abstracts were independently screened for eligibility by two authors (SV, MB). Any reservations on eligibility for inclusion were resolved by discussion between the three reviewers (SV, MB, CC), with final adjudication from FMF. References in English and French provided by colleagues working in the field, in addition to references within reviews of specific techniques, and those in all included articles, were examined to identify additional relevant articles. We included all studies evaluating the performances/abilities of portable devices to assess any aspect of the quality of pharmaceutical products in a laboratory environment, in field surveys and proof-of-concept articles in which the authors stress the potential portability of a method. Studies with the aim to estimate medicine quality prevalence were only included if theycontained information on the performances/abilities of the device as a portable technology for field use. Devices currently under development (although not yet marketed) and devices no longer marketed but superseded by other devices were included. Non-portable devices, devices used for testing the quality of non-pharmaceutical products or for identification of traditional medicines, devices for measuring APIs in biological fluids, and product security technologies were excluded. Patent application publications, articles on the development of a method (eg, a new thin-layer chromatography (TLC) method) not intended for deployment in a field detection kit, reviews/general discussions and articles describing or comparing methods for spectral analysis (chemometrics) rather than the performance of the device itself were also excluded.

CC, MB and SV independently reviewed and extracted data from the eligible articles. For included devices, additional information on objective characteristics (eg, physical appearance, approximate cost and market status) was obtained from manufacturers’ websites and enquiries to them.

### Key variables and definitions

In this review, ‘portable’ refers to transportable equipment (ie, intended to be moved from one place to another whether or not connected to a main electrical supply^13^) able to be carried by a maximum of two persons and that requires minimal set-up on arrival at the field detection site (set-up can be managed by technician-level staff after short training on the device). Devices that require an initial laboratory phase set-up from highly trained staff (eg, Raman spectrometers which require creation of reference libraries and complex processing of spectral data) but that are subsequently portable and easy to use in the field by technician-level staff were included. ‘Field-tested’ device refers to a device assessed onsite, that is, near where the medicines were collected, as opposed to formal laboratory-based studies. A reference standard refers to a specimen of the medicine API intended for use in compendial methods, which is of the highest possible purity and highly characterised by analytical chemistry techniques, used as a direct chemical comparator or to generate a signature.^14^ A reference library refers to a library of measurements of authentic medicines collected by the device and with which the device compares the measurement obtained from a test sample, most commonly spectral libraries of authentic measurements stored within the spectrometer software (‘Spectral Reference Library’). Semi-quantitative is defined as an approximate measurement of the amount of a substance, between a qualitative and a quantitative result (eg, between 80% and 100% of the stated amount). ‘Non-destructive’ refers to devices used to test intact dosage units of medicines (predominantly tablets) either through packaging or without perturbing the dosage unit.

Sensitivity is defined as the proportion of medicines that are detected as poor quality by the device out of all the medicines determined as poor quality by a reference technique. Specificity is defined as the proportion of medicines that are identified as authentic by the device out of all the medicines determined as good quality by a reference technique.

### Data analysis

Data were extracted and entered into a Microsoft Excel spreadsheet. For each device, the developer’s names, type of technology used, main technical specifications (eg, resolution, spectral range), reported sensitivity, specificity and other laboratory or field test results, practical aspects of the use of the device (eg, the measurement time per sample, consumables required), and the pluses and minuses quoted by the authors were extracted when available. For clarity, we have presented only the key results from devices tested on finished pharmaceutical products and only when poor quality medicines (either field-collected or simulated products) were used for evaluation. The quality of the included studies could not be objectively assessed because of the wide heterogeneity of study designs and a lack of consensus guidelines for reporting.

## Results

Of the 5718 reports screened, 282 full-text papers were assessed for eligibility (online supplementary file 3, PRISMA flow diagram). Of these, 62 matched the inclusion criteria and were included in the review.

10.1136/bmjgh-2018-000725.supp3 Supplementary data

**Figure 1:**
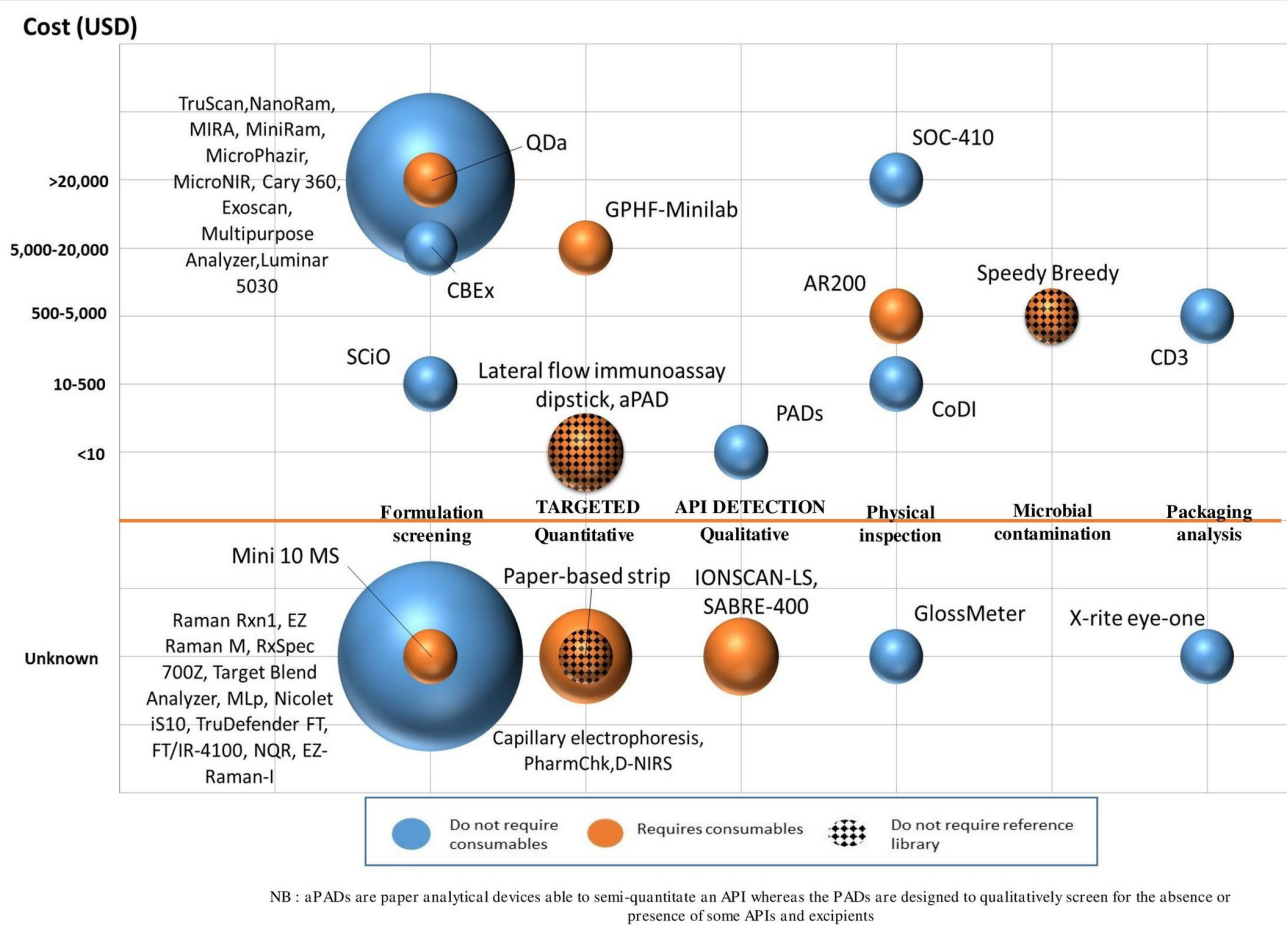
Main characteristics of the included devices by type of analysis, cost at purchase, requirement for consumables and/or reference library. The size of the circles is proportional to the number of devices. APIs, active pharmaceutical ingredients; CoDI, Counterfeit Drug Indicator; PADs, paper analytical devices.

Forty-one devices were identified in the 62 articles (table 1, figure 1).

**Table 1:** Main characteristics of portable devices included in the literature review

Technology	Name of the device (developer)	Market status*†	Approximate purchase cost (US$)†	Handheld‡	References
Raman	TruScan RM (Thermo Scientific, previously Ahura)	M	>20 000	Y	26 27 87§,17 20 78§,15 16 18 19 21 28–30 40 88
	*FirstDefender TruScan (Thermo Scientific)*	*N-Superseded by TruScan RM*	*–*	Y	22
	NanoRam (B&W Tek)	M	>20 000	Y	43 44
	MiniRam II (B&W Tek)	N-Superseded by i-Raman (B&W Tek)	NA (i-Raman: >20 000)	N	26
	MIRA (Metrohm)	M	>20 000	Y	23
	Raman Rxn1 Microprobe (Kaiser Optical)	M	Unknown	N	38
	EZRaman-I (TSI)	M	Unknown	N	24
	EZRaman M Analyzer (Enwave Optronics)	Unknown	–	Y	39
	CBEx (Metrohm Raman)	M	5000–20 000	Y	42
NIR-Fourier transform	MicroPhazir (Thermo Scientific)	M	>20 000	Y	28–31
	*Phazir RX (Polychromix)*	*N-Superseded by MicroPhazir (Thermo Scientific)*	*NA*	Y	26 27§, 89§, 32
	*Phazir RX (Thermo Scientific)*	*N-Superseded by MicroPhazir (Thermo Scientific)*	*NA*	Y	22
	Luminar 5030 (Brimrose)	M	Unknown	Y	26
	Target Blend Analyzer (Thermo Scientific)	M	Unknown	N	26
	MultiPurpose Analyzer (Bruker Optics)	M	Unknown	N	41
NIR-dispersive	MicroNIR (JDSU)¶	M—taken over by Viavi Solution	>20 000	Y	34 90
	D-NIRS (School of Science and Technology, Kwansei Gakuin University)¶	D	Unknown	N	58 59
	SCiO (Consumer Physics)	M	10–500	Y	35
	RxSpec 700Z (ASD)	N-Superseded by other technologies from ASD	Unknown	N	26
MIR-Fourier transform	MLp (A2 Technologies)	N-Superseded by 4500 Series Portable FTIR (Agilent Technologies)	Unknown	N	26
	Nicolet iS10 (Thermo Scientific)	M	Unknown	N	26
	Exoscan (A2 Technologies)	N—now commercialised by Agilent (Exoscan 4100)	>20 000	Y	26
Combined NIR/MIR-Fourier transform	TruDefender FT (Thermo Scientific)	M	Unknown	Y	22
	FT/IR-4100 (JASCO, Tokyo, Japan)	Superseded by FT/IR-4600 (JASCO)	Unknown	N	40
	Cary 630 (Agilent)	M	>20 000	N	29 30
TLC, colourimetry, disintegration test	GPHF-Minilab (Global Pharma Health Fund EV)	M	5000–20 000	N	27 81 87 91§, 92§, 15 46 47 49§, 48
Camera system with various LED sources	CD3/CD3+ (Counterfeit Detection Device version 3/3+) (US FDA)¶	D	500–5000	Y	15 64–66
Lateral flow immunoassay dipsticks	Unnamed (China Agricultural University, Beijing and University of Pennsylvania)¶	D	<10	L	51–53
Paper-based devices	PAD (Paper Analytical Devices) (University of Notre Dame)¶	D	<10	L	60 61
	aPAD (iodometric titration on paper card)¶ (University of Notre Dame)	D	<10	L	56 57
	Paper-based microfluidic strip (unnamed)¶ (Oregon State University)	D	Unknown	L	54
Ion mobility spectrometry	IONSCAN-LS (Smiths Detection, Danbury)	M	Unknown	N	62 63§
	SABRE 4000 (Smiths Detection, Danbury)	M	Unknown	Y	62
Capillary electrophoresis	Unnamed (Hanoi University of Science)¶	D	Unknown	N	55
Reflectance	SOC-410 Directional Hemispherical Reflectometer	M	>20 000	Y	69
	Gloss meter—unnamed (University of Eastern Finland)¶	D	Unknown	Y	93
Microfluidics with luminescence detection	PharmaChk beta 1.1 (Boston University)¶	D	Unknown	N	50
Mass spectrometry	Mini 10 mass spectrometer (Purdue University)	D	Unknown	Y	37§
	QDa single quadrupole (Waters)	M	50 000	N	36
Nuclear quadrupole resonance (NQR)	Unnamed (King’s College, London)¶	D	Unknown	N	45
Reflectance colour measurement	X-Rite Eye-One (Regensdorf)	M	Unknown	Y	67
Low-cost laser absorption/fluorescence	CoDI (Counterfeit Drug Indicator) (Centers for Disease Control and Prevention)	D	10–500	Y	65
Refractometry	AR200 digital refractometer (Leica Microsystems)	M	500–5000	Y	68§
Pressure changes measurement (respirometer)	Speedy Breedy (Bactest)	M	500–5000	N	71

Devices in italics have been superseded.

*D, under development; M, marketed; N, no longer marketed.

†Information from manufacturer website or direct contact with manufacturer.

‡Y, yes; N, no; L, lab-on-a-chip or disposable device.

§Indicates paper published before 2010.

¶Indicates devices for which all articles found in our review were written by author(s) not independent from the manufacturer/developer.

FDA, Food and Drug Administration; LED, light-emitting diode; MIR, mid-infrared; NA, not available; NIR, near-infrared; TLC, thin-layer chromatography.

All evaluations were performed in a laboratory setting unless stated otherwise. We classify devices into those (1) that examine the pharmaceutical formulation, that is, both API(s) and excipients present in the finished pharmaceutical product (‘formulation screening’); (2) those which focus on API(s) detection only; (3) those ‘Physical Analysis Devices’, which primarily assess the physical, rather than chemical, properties of samples; and (4) those that have the ability to detect microbial contamination.

A summary of the reference requirements per device is available in online supplementary file 4, and all the extracted information is presented in online supplementary files 5 and 6.

10.1136/bmjgh-2018-000725.supp4 Supplementary data

### Formulation screening devices

The devices in this section examine the chemical ‘fingerprint’ of a formulation (both API(s) and excipients) and are classified by whether they have been tested for their ability to perform quantitative API analysis or not. The ability of these devices to discriminate between poor quality and good-quality medicines, and to quantitate APIs, depends on both the performance of the device and on the postacquisition processing of spectral data by the associated software. All require a spectral reference library, but are typically non-destructive and do not require consumables.

#### Devices tested for their ability to do quantitative analysis of APIs

As far as we are aware, the devices tested for their abilities to quantitate APIs do not currently have inbuilt software to provide quantitative results. The performance results regarding quantitative abilities of the devices presented in this section were obtained after data processing within the laboratory settings was performed.

The Raman *TruScan RM* (Thermo Scientific) is one of the six devices tested in the field (table 2). It discriminated between 14 poor quality (falsified and degraded medicines) and 70 authentic antimalarials with 100% sensitivity and 99% specificity.^15^ Forty-four falsified samples (of 8 different products) and 62 formulations of genuine products (unstated APIs, 33 ‘product families’ in total) were identified with 100% accuracy.^16^ The TruScan showed similar match/fail performance for medicines identification (despite lower signal resolution) when compared with Raman benchtop instruments.^16–18^

**Table 2:** Main characteristics and performance results of the field-tested devices

Name of the device (developer)	Field test location(s)	Therapeutic indication tested	Reported sensitivity	Reported specificity	Other information and user skill level required
GPHF-Minilab (Global Pharma Health Fund EV)	Gabon, Angola, Brazil, Cameroon, China, Democratic Republic of Congo, Egypt, Ethiopia, Ghana, India, Kenya, Nigeria, Russia, Rwanda, Thailand, Turkey, Uganda, Tanzania, Zambia, Bolivia, Brazil, Colombia, Ecuador, Guyana, Suriname, Venezuela.	Antimalarials^15 46 47 92^; antibiotics.^92^	29% of extremely non-compliant samples*† for both content and dissolution correctly identified by the Minilab^47^; Se‡ for both ID test and content test=79%, Se‡ for ID test only=100%.^15^	Sp‡ for both ID test and content test=100%, Sp‡ for ID test only=100%.^15^	Visual appearance did not provide consistent results when performed by MRA staff or lab staff.^47^ Some lab skills required—at least 1-week training and proficiency testing recommended.
CD3/CD3+ (US FDA)	Ghana.	Antimalarials.^15^	Se‡=100% for analysis based on packaging materials and dosage unit,^15^ Se‡=100% for dosage unit-only analysis.^15^	Sp‡=53% for analysis based on packaging materials and dosage unit,^15^ Sp†‡=64% for analysis dosage unit-only analysis.^15^	More reliable to conduct side-by-side comparisons with physical authentic samples than using the library images CDAIL.^15^ Low skill level required—performances increase with experience.
Lateral flow dipstick immunoassay (Unnamed)	Colombia, India, Papua New Guinea, Zambia.	Antimalarials.^51^	NA—no gold standard reported.^51^ NB: 2%–4% cross-reactivity of artemether and artesunate to artemisinin.^51^	NA—no gold standard reported.^51^	NA—no gold standard reported.^51^ Low skill level required.
TruScan RM (Thermo Scientific)	USA.	Erectile dysfunction drug.^19^	Testing by unknown number of special agents unfamiliar with instrument and procedure: Acc§ for identification of the presence/absence of sildenafil (n=14)= 91.7%.^19^		A sample preparation (extraction, filtration, addition of silver colloid) was performed, the obtained sample solution was then tested in a phial.^19^ Training of 20 min considered as sufficient (test by four analysts only).^19^
CBEx (Metrohm Raman)	India, Zimbabwe.	Antimalarials, antibiotics and others (not detailed in the publication).^42^	NA.¶		Rugged (instrument dropped accidentally twice with no observed altered functioning; no problem during routine international air transportation and travel by vehicle to various sites; instrument withstood temperatures between room and 40°C temperatures.^42^ Less than 2 weeks training estimated as sufficient to become basic to advanced user.^42^
Speedy Breedy (Bactest)	India, Zimbabwe.	Antibiotics, sterile sodium chloride, purified water.^71^	NA.¶		Long time run=power interruption required to restart the run of the sample the next day; biological waste management required; carry case not robust enough at the time of study.^71^ Less than 2 weeks training estimated as sufficient to become basic to advanced user.^71^

*Extreme deviation was defined as a deviation of 20% or more from the declared amount of API as determined by assay, and/or a percentage of active ingredient dissolved 25% or more below the pharmacopoeial limit Q in dissolution testing.

†Against HPLC and dissolution testing (please note that disintegration testing is not an appropriate proxy for dissolution testing).

‡Against HPLC analysis.

§Against FT-IR

¶Field evaluation aimed at testing the field utility of the device rather than its performance.

Acc, Accuracy: API, active pharmaceutical ingredient; CDAIL, CD3 Authentic Image Library; HPLC, high-performance liquid chromatography; ID, Identification; MRA, medicines regulatory authority; NA, not available; Se, sensitivity; Sp, specificity.

After applying a sample preparation method, special agents in a mail facility tested 14 samples of Viagra (12 contained sildenafil) with the TruScan RM with an accuracy of 91.7% to qualitatively identify the presence/absence of sildenafil (Fourier Transform-Infrared (FT-IR) analysis as reference technique).^19^ Different strengths (simulating ‘substandard’ medicines) of the same antimalarial APIs and brand could not be reliably distinguished using the match/not-match approach.^15 20^ In addition, one in three placebos wrongly passed the identification test versus their full API strength counterpart.^16^ In investigations of the p values (threshold limit for a sample to give a ‘pass’ or ‘fail’) obtained by scanning five products containing candesartan with the TruScan RM, it was suggested that the p value could be set at 0.40 (instead of 0.05) for the device to better discriminate substandard medicines containing less than 50% or more than 150% API from the good-quality products.^21^ However, these results should be taken with caution considering the small sample size. The FirstDefender TruScan (superseded by the TruScan RM) determined the amount of APIs to within 1.6%–12% of the reference assay for experimental finished products of acetylsalicylic acid, ascorbic acid and caffeine.^22^

The *Metrohm Instant Raman Analyzer* (MIRA, Metrohm) discriminated between different concentrations of injectable doxorubicin (n=90) and epirubicin (n=90) through glass containers with 100% sensitivity and specificity. Quantitation with a coefficient of determination (r^2^) of 0.99 was reported.^23^

The *EZRaman-I* (TSI) qualitatively confirmed the presence of the stated API in four finished drug products containing amoxicillin, acyclovir and doxycycline^24^ with a Raman binary barcode method using a reference library containing Raman spectra of APIs.^25^ On averaging the results of the per cent of the API claimed on the label of five tablets per sample, predictions by the EZRaman-I were within 3% of the HPLC results for three out of four products, and one product showed a value of 6.0% lower than those obtained by HPLC.

Among the 14 devices based on Infrared (IR)/Mid-Infrared (Mid-IR), the *MicroPhazir* (and its predecessor the Phazir) has been tested on small sample sets of different types of raw or finished pharmaceutical product types in the laboratory.^22 26–33^ Spectral data have been successfully acquired through transparent blister packaging using six experimental samples.^28^ Quantitation of acetylsalicylic acid, ascorbic acid and caffeine in tablets gave results comparable with a reference benchtop Fourier Transform-Near-Infrared (FT-NIR) instrument.^22^

The *MicroNIR* (JDSU, then Viavi) and the *TruDefender FT* (Thermo Scientific) have been used for quantitation of API to within 0.1%–7.8% of the reference for weight loss and erectile dysfunction medicines, and also for acetylsalicylic acid, ascorbic acid and caffeine.^22 34^

The *SCiO* (Consumer Physics) device showed 100% specificity and sensitivity in the identification of falsified (n=42) versus genuine (n=54) antimalarials, but failed to quantify the amount of amodiaquine in finished products. This device was able to quantify artesunate with 95% certainty in 15 oral products.^35^

The compact benchtop *QDa single quadrupole mass spectrometer* (Waters) correctly identified the Artemisinin-based combination therapy (ACT) artemether+lumefantrine and other compounds, such as chloramphenicol, ciprofloxacin and sugars, in 192 seized falsified antimalarials. The relative intensity of each compound detected could be compared from run to run between tablets and used as a proxy for API quantitation.^36^ For quantitation, the handheld *Mini 10* mass spectrometer has been demonstrated to produce parts-per-billion detection limits for drugs of abuse 37 but has not yet been evaluated for poor quality medicines.

#### Devices with untested potential to perform quantitative analysis of APIs

Three Raman devices (MiniRam II (B&W Tek), Raman Rxn1 Microprobe (Kaiser Optical) and EZRaman M Analyzer (Enwave Optronics))^26 38 39^ and nine near-infrared/mid-infrared devices (MultiPurpose Analyzer (Bruker Optics), Luminar 5030 (Brimrose), RxSpec 700Z (ASD), Exoscan (A2 Technologies), MLp (A2 Technologies), FT/IR-4100 (JASCO), Cary 630 (Agilent), Nicolet iS10 (Thermo Scientific) and Target Blend Analyzer (Thermo Scientific)) were included in small-scale laboratory studies.^26 29 30 40 41^

Of note, the *CBEx* (Metrohm Raman) successfully identified the presence of paracetamol, amoxicillin, lumefantrine and pyrazinamide in various APIs/API combinations but failed to identify other APIs in fixed-drug combinations, (eg, artemether in artemether-lumefantrine tablets), and furosemide and oxytocin in single API injection samples.^42^ A limited set of artificially degraded samples were correctly identified with accuracies depending on the API and the level/type of degradation. A field evaluation of the utility of the device, rather than its performance (table 2), among 10 operators from the regulatory authorities of India and Zimbabwe with various technical experience, suggested this as a well-functioning device requiring less than 2 weeks of training.^42^

The *NanoRam* (B&W Tek) showed 100% sensitivity and 96% specificity (against TLC, with HPLC used to confirm samples which failed TLC) in the investigation of 289 antimalarial samples (including 24 falsified and 22 experimental ‘wrong API fakes’ containing paracetamol).^43 44^

A prototype *nuclear quadrupole resonance (NQR) device* has been used successfully to identify ampicillin in capsules and paracetamol in tablets through their original packaging. No data on its sensitivity and specificity have yet been reported.^45^

### Targeted API detection devices

Most devices in this section are semi-quantitative. All techniques require sample destruction and most require sample pretreatment (eg, dissolution). Some provide both qualitative and quantitative information and may provide data on other properties (eg, disintegration characteristics). Others simply qualitatively identify the API.

#### Quantitative and semi-quantitative targeted API detection devices

The *GPHF-Minilab* (Global Pharma Health Fund) is a ‘lab-in-a-suitcase’ containing the supplies for visual physical inspection of the medicine (both dosage form and packaging), identification and semi-quantitation of the stated API by TLC, and disintegration testing. It is one of the six field-tested devices identified in this review (table 2).

In a field survey of 84 antimalarial medicines (including 14 substandard/falsified samples) in Ghana, 100% sensitivity and specificity were reported for Minilab TLC identification against HPLC reference assays.^15^ For API identification and semi-quantitation by TLC, the sensitivity was 79% with 100% specificity. In Brazil, 14 of 46 (30%) quinine samples were judged substandard by Minilab TLC with semi-quantitation. However, the seven samples that underwent confirmatory tests were all found to be within specifications.^46^ In the same study, all 289 samples collected in Guyana passed TLC with semi-quantitation, but 5 out of 10 samples failed subsequent confirmatory testing. A multicountry survey in Africa found that the Minilab detected 30% of 31 very non-compliant (deviation of >20% from stated API by HPLC and/or percentage of API dissolved >25% below the pharmacopeial limit Q in dissolution testing) antimalarial samples.^47^ However, dissolution and disintegration tests measure different aspects of a solid formulation and we would not expect full agreement. TLC testing failed to identify the 77 substandard antimicrobials, among which 76 samples contained %API >80% and <123% of the label claim (specificity of 97%).^48^ However, the Minilab is designed to detect samples with API below the 80% threshold. Interobserver variability was identified as a significant contributor to Minilab semi-quantitative inaccuracy.^46 49^ In Tanzania, seven drug inspectors assessed finished products containing four different APIs (antibiotics and antimalarials) at three different concentrations (0%, 40%, 100%).^49^ Twenty-five out of 28 substandard samples with 40% API were incorrectly identified by TLC as of acceptable quality. After further training, 8 out of 28 samples were still wrongly identified as of acceptable quality. All samples with 100% API, zero API and with wrong API were correctly identified.

The *PharmaChk* is a field-portable microfluidics device, currently limited to artemisinin-based drugs, designed for API quantitation and tablet dissolution testing.^50^ Quantitation of three oral artesunate formulations (mean of five samples per formulation) showed accuracy to within 0%–4% of HPLC values.^50^

Single-use *lateral flow immunoassay dipsticks* (resembling rapid malaria diagnostic tests in appearance) use monoclonal antibodies to detect poor quality artemisinin-based antimalarials.^51–53^ Field survey samples (table 2) were not tested against a reference technique and hence sensitivity or specificity cannot be calculated.^51^ In laboratory testing, artesunate dipsticks showed 100% specificity for detecting artesunate against other commonly used antimalarials, including other artemisinin derivatives.^53^ A semi-quantitative analysis of API content was obtained by sample serial dilutions.^53^

A proof-of-concept paper describes the adaptation of the Fast Red TR reaction for artesunate detection onto *paper-based microfluidic strips*.^54^ The cards could detect the presence and determine relative concentration of artesunate in one genuine sample, and could detect its absence in two formulations containing artemether and dihydroartemisinin. Semi-quantitation accuracy was improved by greyscale intensity analysis using a smartphone app.

A battery-powered *capillary electrophoresis* device was able to successfully identify and quantify salbutamol and metoprolol in syrup and tablet formulations. Quantitation accuracy was within 3%–13% of results obtained by HPLC.^55^

Paper cards (*aPAD*) have been successfully used for semi-quantitative *iodometric back-titration* of amoxicillin and ampicillin, tests specified in the pharmacopeial analysis of β-lactam antibiotics.^56 57^ These cards differentiated between amoxicillin solutions that varied in concentration by 0.15 mg/mL, allowing identification of substandard amoxicillin <83% of labelled API content.^56^ The aPADs gave errors of semi-quantitation of 13% and 5% (compared with HPLC) for 41 samples of amoxicillin and 40 samples of ampicillin collected in Kenya, respectively.^57^ In that study, aPADs identified samples containing below/above the US Pharmacopeia 90.0% limit of the medicine stated %API, with sensitivities of 73.2% and 80.0% for amoxicillin (n=80) and ampicillin (n=56), respectively (100% specificities). The authors suggested that artificially degraded samples made for the purpose of the study (thermally stressed) may have led to decreased sensitivities.

By obtaining two-dimensional spectral data of tablets, an *NIR imaging device (D-NIRS)* could evaluate the distribution of different chemical components during tablet dissolution,^58 59^ but no sensitivity and specificity data of this device are available.

#### Purely qualitative targeted API detection devices

In contrast to the aPADs that enable semi-quantitation based on one chemical reaction (see above), the *paper analytical devices (PADs)* are designed for qualitative screening of APIs and some excipients.^56 60 61^ Separate lanes housing different colourimetric reactions produce a ‘colour bar code’, which is compared with a reference library of ‘standard colour bar code images’. Expert readers can even discriminate different strengths of APIs.^61^

In testing of experimental formulations of known concentration, two antibiotics, three antituberculosis medicines and two antimalarials produced sensitivity values of 76%–100% (n=9–60) and specificity of 80%–100% (n=30–135).^60 61^ The identification of APIs in coformulated samples was more variable. For example, in testing coformulated tuberculosis (TB) medicine samples, ethambutol was not detected when actually present in 30% and falsely reported as present when absent in 17% of tests.^60^ In testing 30 two-API coformulated TB samples, ethambutol and isoniazid were correctly detected in all samples.^60^

Two *ion mobility spectrometry devices* have been evaluated.^62 63^ The *IONSCAN-LS* (Smiths Detection) detected the APIs of erectile dysfunction drugs in 26 herbal supplements with 100% sensitivity and specificity, with successful identification of the specific API in 13 of 15 (87%) samples.^63^ The *SABRE 4000* (Smiths Detection) showed comparable results with a benchtop ion mobility instrument in detecting sibutramine in dietary supplements.^62^

### Devices which primarily examine physical properties

Devices in this section primarily examine physical properties of the sample, such as their visual appearance. They cannot verify the presence or absence of the API. As falsified packaging is the key for identifying falsified medicines, they may have an important parallel functionality to chemical analysis devices.

#### Visual/colour inspection

The *Counterfeit Detection Device CD3* **+** unmasks differences between test and authentic samples (packaging and dosage forms) by allowing the user to compare their appearance under diverse ultraviolet-visible and infrared (IR) wavelengths.^15 64–66^ With this technique, falsified and genuine artesunate blister pack samples (n=203) were identified with sensitivity and specificity of 98.4% and 100%, respectively, with 100% interuser reliability.^64^ In a field study (table 2), 84 artemisinin-based combination therapies were identified with a sensitivity of 100% and specificities of 53% (on packaging materials and dosage unit) and 64% (on dosage unit only).^15^

The *X-Rite Eye-One* is an optical spectrometer that projects light of wavelengths 380–730 nm towards a solid surface, collects the reflected visible spectrum and digitally records it for comparison with a reference genuine sample.^67^ It correctly identified 40 out of 41 (98%) samples of erectile dysfunction medicines, among which 35 were falsified. However, on testing genuine samples, 25% of packages and 15% of tablets were wrongly identified as falsified.

#### Other physical properties

Refractometry can be used to quantitatively detect APIs in solution by comparing the measured concentration with a concentration curve constructed from known standards. In testing whether an API was within 80%–120% of the stated concentration, the *AR200 digital refractometer* (Leica Microsystems) showed a sensitivity of 83%–100% and specificity of 56%–87% for 458 samples of 5 different poor quality antimalarials (both tablets and injectables).^68^

The *Counterfeit Drug Indicator* (CoDI) measures the ratio of laser light intensity transmitted and scattered on passing through a tablet, and compares the result from the test sample with that from an authentic tablet. The device correctly discriminated 6 falsified and 12 authentic artemether-lumefantrine tablets.^65^

The handheld *SOC-410* (Surface Optics Corporation) uses directional hemispherical reflectance to analyse the surface of tablets in the mid-infrared and near-infrared range without the need for complex spectral interpretation. It showed 100% accuracy in the identification of one genuine and four falsified Viagra samples.^69^

A handheld *gloss meter,* based on diffractive optical elements, was developed to analyse differences in the magnitude of specular gloss of the surface of authentic and falsified tablets. The device results showed consistency with the findings from a two-dimensional gloss meter and an optical interference profilometer to screen for two falsified artemether-lumefantrine samples from Ghana.^70^

### Microbial contamination detection

The *Speedy Breedy* (Bactest), a portable respirometer that detects pressure changes as a proxy of microbial growth and hence contamination in liquid samples, showed sensitivities from 93.0% to 100% and specificities of 100% to identify microbial contamination by *Escherichia coli* of samples of sterile water for injection that were purposively spiked under various laboratory experimental conditions. Artesunate and oxytocin products for injection were correctly characterised for the presence/absence of microbial contamination with *E. coli* and *Pseudomonas aeruginosa.* Further evaluation of the field utility of the device (rather than its performance) showed that, despite the ability of the device to generate results under uncontrolled field settings (India and Zimbabwe), the requirement for a continuous power source during analysis (which can take more than a day) might be a barrier to its use in remote settings (table 2).^71^

## Discussion

The above results demonstrate the huge diversity of technologies and devices becoming available for the field detection and evaluation of medicines.

To maximise the detection and removal of poor quality medicines from the supply chain, a screening device with high sensitivity is required. Specificity is less vital as although low values would lead to additional work and cost in reference laboratory assays they would not lead to patient harm. Sensitivity data were found for few devices and were mostly derived from results of laboratory testing on a small number of samples of a few APIs. The median (range) number of APIs that were assessed per device was only 2 (1–20), a very meagre proportion of the ~7000 global international non-proprietary names of pharmaceutical substances.^72^

The increasing sophistication of falsified medicines requires advanced techniques that detect anomalies of packaging or product not apparent to the naked eye. Of the included devices, the CD3 and the X-Rite showed high sensitivity for packaging authenticity evaluation. The low-cost single-use technologies showed promise for qualitative analysis (PADs, lateral flow immunoassay dipsticks) and could be of great interest in the distal pharmaceutical supply chain. Very few devices have been evaluated for their ability to distinguish genuine from substandard medicines with reduced %API. Most devices with the potential to assay API (semi)quantitatively in finished products require consumables and are destructive (GPHF-Minilab, PharmaChk, aPADs, lateral flow immunoassay dipsticks, paper-based microfluidic strip and capillary electrophoresis), except for spectroscopic devices. However, of the nine spectroscopic devices (TruScan, MIRA, EZRaman-I, MicroPhazir, MicroNIR, TruDefender, SCiO, QDa and Mini 10 mass spectrometer) tested for quantitation, none used automated methods, but required highly trained operators using complex API-specific calibration models, and are therefore not field-ready.

Tablet dissolution characteristics are key determinants of bioavailability and therefore efficacy. No marketed portable devices are currently able to evaluate dissolution, despite the likely contribution of poor dissolution antimicrobials to AMR.^10^ The under-development D-NIRS was the only portable device assessed for its ability to monitor dissolution and showed promising results, although on a limited number of samples. Methods for detecting other quality defects in substandard medicines, such as the presence of impurities or the lack of sterility, have received very little attention, except the Speedy Breedy which recently showed promise to identify microbial contamination in liquid samples.^71^ The distinction between degraded medicines, which left the factory with good quality but deteriorated due to poor storage and transport, and those failing due to errors in factory production is vital as the origins and solutions are different. The development of reference and screening API-specific technologies that could distinguish these issues will be of great importance.

Two-thirds of the devices (27/41, 66%) identified use spectroscopic techniques. Of these, only the TruScan and the NanoRam have been tested on a large number of samples in the laboratory. However, it was not possible to reliably and comprehensively review devices such as the TruScan for their performance, because one of the key publications could not be evaluated since the APIs contained in the products tested were not detailed.^16^ This emphasises the importance of ensuring that the databases of the results of device evaluations are made available to ensure their enduring value. A major advantage in an L/MIC setting of many of the spectroscopic devices is the need for minimal end-user training, provided that the chemometrics analysis steps have already been bundled in user-friendly software. However, there are obstacles to their implementation. First, the purchase cost of most of these devices is high, likely prohibitively so, in L/MIC settings. User-friendly, miniaturised, low-cost spectrometers such as the SCiO (which can be operated using a smartphone) have recently shown promising performance.^35^ Second, the need for up-to-date reference specimens, whether as a prestored ‘spectral reference library’ or physical samples of quality-assured genuine medicines, adds significant work.^73^ ‘In-built’ libraries of raw materials available in some spectrometers are inappropriate for the screening of finished pharmaceutical products, as the spectra obtained are often influenced by both APIs and excipients and vary between brands. The difficulty of assembling quality-assured comparators and the need for frequent updating of stored spectral signatures may present a barrier to use unless the pharmaceutical industry efficiently and promptly provides updated samples or spectra when manufacturing processes change. Almost 30% (n=114) of antimalarials collected in one study could not undergo Raman analysis because the authentic comparators could not be obtained by the investigators.^43^ Discussion on industry standards for spectra file format and transferability between devices using the same technology will be important.

Each spectroscopic technique has unique advantages and disadvantages. Using a combination of different spectroscopic techniques in parallel may be beneficial. For example, using a Raman spectrometer in combination with an IR spectrometer for tablets containing relatively low quantities of APIs may improve detection.^16 44^ Combining a spectroscopic tool with a visual inspection tool may also be synergistic. As far as we are aware, there have been no evaluations of combined technologies.

The widely distributed GPHF-Minilab (more than 800 units distributed in 95 countries)^74^ showed good performance in the identification of falsified medicines by TLC in one study and consistently high specificity. Results were user-dependent, underlying the importance of regular good quality training and proficiency testing. Of note, the disintegration testing in the Minilab kit is not an appropriate proxy for dissolution testing. The device showed limited ability to identify substandard medicines. In a recent study in China in which 77 samples were substandard, most being above the 80% API threshold in HPLC testing, 0% sensitivity (97% specificity) was reported.^48^ However, vitally, the Minilab does not claim to be able to detect substandard medicines with API content above the 80% limit. Its main function has been to detect zero and wrong API medicines.

There are important limitations to this review. Our search included only scientific databases and only in English. We discounted 29 articles in which the stated aim was to develop, validate or compare chemometric techniques, rather than to assess the performance of the portable device itself, thereby excluding 13 portable devices (online supplementary file 7). This includes the Matrix-F (Bruker Optics), a non-handheld device used in over 300 mobile laboratories in China.^75^ Findings of device evaluation performed by non-independent evaluators risk bias and should be interpreted with caution.

10.1136/bmjgh-2018-000725.supp7 Supplementary data

Comparison between devices was significantly hindered by the heterogeneity of device evaluation methods and reporting. We found only two studies in which the Standards for Reporting Diagnostic Accuracy studies were followed.^43 48^ Standardised guidance on how to assess and compare the performance of screening devices would be of great benefit. A recent article from the US Pharmacopoeial Convention addresses this.^76^ There is an urgent need for international organisations, the device and pharmaceutical industry and regulatory authorities to discuss norms and standards for medicine quality screening devices. In addition, field testing was conducted for just six devices, leaving a paucity of data on performance in the ‘real-world’.

Other key gaps in the literature were identified (box 3). We observed a dire lack of information as to which medicines and formulations can be evaluated with each device. There has been a focus on anti-infective medicines (especially antimalarials), neglecting other medicine classes.

Box 3 **Key gaps in the literature**

- Lack of independent evaluation of the majority of devices, particularly in field settings.
- Device performance tested on a very limited subset of available active pharmaceutical ingredients, predominantly anti-infectives.
- Very limited testing and comment on the ability of the devices to test through packaging and the type of packaging that is least obstructive to device use.
- Very limited comment on the inability of Raman or infrared spectroscopy to test capsules non-destructively, due to the opacity of capsule coating.
- Very limited information on the performance of devices to test liquid or parenteral formulations is available, with no data on testing of topical formulations.
- No studies looking at the effect of tablet coating on device performance.
- No information on cost-effectiveness.
- No testing or comment on the ability of the devices to distinguish between chiral enantiomers.
- Very limited comment on where in the pharmaceutical supply chain which devices are best employed.
- Very few studies which comment on training needs for accurate use of the devices.

Chemical structures suggest a priori that some APIs will be problematic for certain devices. For example, NQR can only detect APIs with quadrupolar nuclei, such as ^14^N. This is present in over 80% of medicines,^77^ but not, for example, the artemisinin derivatives.^62^ Similarly, some APIs, such as artesunate and quinine sulfate, have strong fluorescence with weak Raman scattering at 785 nm, impairing the ability of such Raman devices to detect poor quality products labelled as containing these APIs.^20 78^ Raman scattering from medicines with relatively low amounts of API(s) is often insufficient.^16 44^ Sensitivity of the PADs is also reduced for formulations with a low proportion of API.^61^ More than half of pharmaceuticals are chiral compounds, with many enantiomers of racemic drugs showing marked differences in pharmacology.^79 80^ No discussion was found on the ability of the reviewed devices to discriminate different enantiomers. Theoretically only NQR would have this capability.

Most of the tested finished products in the included studies were tablets. Certain tablet coatings will likely provide a very difficult barrier to optical spectroscopic examination, as shown in testing of blue-coated dihydroartemisinin with the NanoRam.^81^ No data were found on testing of topical applications (eg, creams, gels) and little on liquids. It is unclear whether the devices lack the capability to test these formulations, or simply that this has not yet been investigated.

One vital but undiscussed issue is that (with the probable exception of NQR) it will not be possible to non-destructively evaluate capsules unless spectroscopic techniques can be developed that allow the devices to ‘see through’ the capsule material. Consequently, a very sizeable proportion (in Laos, UK, France and USA, capsules comprise 11.4%,^82^ 17.7%,^83^ 9.7%^84^ and 7.7%,^85^ respectively, of registered oral medicines) of the global medicine supply will not be amenable to non-destructive spectroscopic evaluation. Non-destructive sampling was highlighted by different regulatory authority stakeholders as an ideal feature of a medicine quality screening device in a recent qualitative research paper.^73^ The use of transparent capsule shells could greatly expand IR or Raman devices utility. There are also few data on the ability of devices to accurately assess medicines through packaging (important when sample size is small and samples are required for legal investigations). How spectroscopic device accuracy changes with different types of glass and plastic packaging seems unknown. With such information, blister pack and tablet/capsule/powder/liquid bottle packaging could be designed to facilitate spectroscopic evaluation.

Further key aspects that have received minimal discussion include issues of device maintenance and quality assurance/quality control of the device performance (including calibration and performance quality checks).

The cost-effectiveness of introducing devices within postmarketing surveillance (PMS) systems, compared with other solutions for strengthening PMS, has not apparently been investigated. Screening technologies should be considered within a broader strategy to reduce the risk of poor quality medicines reaching patients. Given the substantial costs of using most of the devices in L/MICs, investment in devices should be compared with other strategies, such as enhancing inspection of manufacturing sites and evaluation of product dossiers. The public health effectiveness of detection of poor quality medicines will not be fulfilled unless reference laboratories are accessible and appropriate rapid responsive action is conducted.

How devices can be optimally used in different parts of the pharmaceutical supply has been little discussed, nor their integration into PMS. Their abilities may be overappreciated and vital routine visual packaging inspection reduced. Non-governmental organisations, procurement agencies and other institutions supplying medicines may also benefit from the use of reliable devices to check the quality of medicines they procure. Because those involved may have diverse educational backgrounds, data are needed to better understand the minimum level of training needed for appropriate use of the devices.

It seems unlikely, with current technology, that one device will be able to effectively monitor the quality of all medicines, and exploration of combinations of devices with different faculties is needed. The synergistic combination of devices with smartphones containing registration, batch number and packaging information for the country’s medicines, and alerts of poor quality medicines in the region and to and from WHO holds great promise. As research expands on screening devices for testing different APIs, especially those coformulated, care will be needed with the public release of these data in order to avoid informing those making poor quality medicines of information that would allow them to circumvent detection of their ‘products’ by the screening devices.

For a small proportion of the globally available APIs, there is evidence that some devices will reliably detect falsified medicines, often containing zero or wrong API. However, there is much less evidence for their ability to detect substandard medicines, usually containing either too much, too little API or impaired dissolution. If such devices are used, it will be important to recognise this issue and not to regard a pass result as meaning that a medicine is good quality, only that it is not falsified. Clear statements from manufacturers and those evaluating these devices on their claimed capabilities and their limitations will be crucial to avoid overconfidence in their abilities.

## Conclusion

The diversity of devices for medicines quality screening holds great hope for empowering medicine inspectors, making their work more cost-effective and actionable, and protecting patients from the harm of poor quality medicines. However, there is a vitally important lack of independent evaluation of the majority of devices, particularly in field settings. There is currently no device demonstrated to be able to screen the quality of all existing APIs available globally in different formulations and in different settings. Training and costs of implementing screening devices are major concerns, especially in L/MICs, but these considerations have not been explored. Intensive research is needed in order to provide the evidence national MRAs need to determine the optimal choice of device(s) to combat poor quality medicines.

10.1136/bmjgh-2018-000725.supp5 Supplementary data

10.1136/bmjgh-2018-000725.supp6 Supplementary data
