# Supplementary material for: Field detection devices for screening the quality of medicines: a systematic review
Source: BMJ Glob Health. 2018 Aug 29;3(4):e000725. doi: 10.1136/bmjgh-2018-000725 (PMC6135480; doi:10.1136/bmjgh-2018-000725)
Supplement: Supplementary data [file bmjgh-2018-000725supp002.pdf]

Supplementary file 2. Search terms per search engine

Embase.com, Pubmed and Web of Science

(technology or technique or device or apparatus or instrument or system or detector or method or analyzer) AND (portable or handheld or battery powered or field test or mobile or rapid) AND ("medicine quality" or "drug quality" or "pharmaceutical quality" or counterfeit* OR fake* OR spurious OR substandard* OR falsified OR "falsely labelled" OR "falsely labeled")

SciFinder

Thirty-six searches were performed in SciFinder, using the main key terms, because all the key terms could not be searched at once.

1. Portable (handheld) device (technique, technology) for medicine quality (drug quality, pharmaceutical quality)
2. Portable (handheld) device (technique, technology) for Falsified medicine (falsified drug, counterfeit medicine)
3. Portable (handheld) device (technique, technology) for fake medicine (substandard medicine)
4. Portable (handheld) device (technique, technology) for fake drug (substandard drug)
5. Portable (handheld) device (technique, technology) for spurious medicine (spurious drug, counterfeit drug)
6. Portable (handheld) device (technique, technology) for falsely labelled medicine (falsely labeled drug)
7. Portable (handheld) instrument (apparatus) for medicine quality (drug quality, pharmaceutical quality)
8. Portable (handheld) instrument (apparatus) for Falsified medicine (falsified drug, counterfeit medicine)
9. Portable (handheld) instrument (apparatus) for fake medicine (substandard medicine)
10. Portable (handheld) instrument (apparatus) for fake drug (substandard drug)
11. Portable (handheld) instrument (apparatus) for spurious medicine (spurious drug, counterfeit drug)
12. Portable (handheld) instrument (apparatus) for falsely labelled medicine (falsely labeled drug)
13. Portable (handheld) detector (analyzer) for medicine quality (drug quality, pharmaceutical quality)
14. Portable (handheld) detector (analyzer) for Falsified medicine (falsified drug, counterfeit medicine)
15. Portable (handheld) detector (analyzer) for fake medicine (substandard medicine)
16. Portable (handheld) detector (analyzer) for fake drug (substandard drug)
17. Portable (handheld) detector (analyzer) for spurious medicine (spurious drug, counterfeit drug)
18. Portable (handheld) detector (analyzer) for falsely labelled medicine (falsely labeled drug)
19. Portable (handheld) method (system) for medicine quality (drug quality, pharmaceutical quality)
20. Portable (handheld) method (system) for Falsified medicine (falsified drug, counterfeit medicine)
21. Portable (handheld) method (system) for fake medicine (substandard medicine)

22. Portable (handheld) method (system) for fake drug (substandard drug)
23. Portable (handheld) method (system) for spurious medicine (spurious drug, counterfeit drug)
24. Portable (handheld) method (system) for falsely labelled medicine (falsely labeled drug)
25. Mobile instrument (mobile apparatus, mobile technology, mobile technique) for medicine quality (drug quality, pharmaceutical quality)
26. Mobile instrument (mobile apparatus, mobile technology, mobile technique) for Falsified medicine (falsified drug, counterfeit medicine)
27. Mobile instrument (mobile apparatus, mobile technology, mobile technique) for fake medicine (substandard medicine)
28. Mobile instrument (mobile apparatus, mobile technology, mobile technique) for fake drug (substandard drug)
29. Mobile instrument (mobile apparatus, mobile technology, mobile technique) for spurious medicine (spurious drug, counterfeit drug)
30. Mobile instrument (mobile apparatus, mobile technology, mobile technique) for falsely labelled medicine (falsely labeled drug)
31. Rapid test (field test) for medicine quality (drug quality, pharmaceutical quality)
32. Rapid test (field test) for Falsified medicine (falsified drug, counterfeit medicine)
33. Rapid test (field test) for fake medicine (substandard medicine)
34. Rapid test (field test) for fake drug (substandard drug)
35. Rapid test (field test) for spurious medicine (spurious drug, counterfeit drug)
36. Rapid test (field test) for falsely labelled medicine (falsely labeled drug)
