# Supplementary material for: Field detection devices for screening the quality of medicines: a systematic review
Source: BMJ Glob Health. 2018 Aug 29;3(4):e000725. doi: 10.1136/bmjgh-2018-000725 (PMC6135480; doi:10.1136/bmjgh-2018-000725)
Supplement: Supplementary data [file bmjgh-2018-000725supp004.pdf]

Supplementary file 4. Reference requirements per device

Table 3. Reference Requirements per Device

Category	Subcategory	Device	Minimum Reference Required	Pre-recorded	Sample needed for reference
**Formulation Screening Devices**	*Quantitative*	Select Raman, NIR and MIR spectrometers	Spectral reference library	M	G
		Mass spectrometry	Reference table of values or spectral reference library.	O	R
	Qualitative. Undemonstrated quantitative ability	Select Raman, NIR and MIR spectrometers	Spectral reference library	M	G
		NQR	Reference table of values or spectral reference library.	O	G
**Targeted API Detection Devices**	*(Semi-)Quantitative*	Minilab	Reference sample (preferably brand specific)	NP	R
		PharmaChk	Reference sample/calibration standard(s) of API.	NP	R
		Lateral Flow Immunoassay Dipsticks	None	N/A	N/A
		Paper-based microfluidic strip	None	N/A	N/A
		Capillary electrophoresis	Genuine brand-specific sample or reference standard	NP	G
		Iodometric back titration	None (positive control needed for performance verification)	N/A	C
		D-NIRS	Genuine sample. (Primarily for dissolution	O	G

			testing purposes).		
	Purely Qualitative	PADs	Library of images	O	R/C
		Ion mobility spectrometry	Genuine sample or reference standard	N/O	G (preferable)
**Devices which primarily examine physical properties**	*Visual/colour inspection*	CD3	Library of images (dosage form and packaging)	O	G
		X-Rite Eye-One	Spectral reference library (dosage form and packaging)	M	G
	Other physical properties	Refractometry	Reference table of values and at least one known solution.	O	G
		CoDI	Reference table of values.	O	G
		SOC-410	Reference table of values	O	G
		Glossmeter (unnamed)	Gloss value	O	G
**Microbial contamination detection**		Speedy Breedy	None (negative control needed)	N/A	N/A

G, Genuine brand-specific sample: a quality-assured genuine sample of the medicine under test, from the same manufacturer and at the same dose.
R, Genuine non brand-specific sample: a quality-assured genuine sample of medicine containing the same API and the same dose as the medicine under test, but which is not manufacturer-specific.
C, chemical reference standard: a highly-characterised, quality-assured specimen of the pure API.
N, None: no reference sample is required, but good practice requires use of a reference sample either brand-specific or non brand-specific as a positive control.
'**Pre-loaded**' refers to the ability to acquire the reference data prior to the time of sample testing:

- **M, Mandatory**: the device requires the uploading of pre-recorded reference data prior to use
- **O, Optional**: pre-recorded reference data can be uploaded prior to use if desired, or the genuine sample can be run at the same time as the test sample for comparison.
- **NP, Not Possible** – the device requires a brand/non brand-specific genuine sample to be run at the same time as the test sample.

A **spectral reference library** consists of previously-recorded spectra of genuine samples.
A **reference table of values** consists of values from previous measurement of the reference, and stored either on the instrument or in a separate location.
