# Supplementary material for: Field detection devices for screening the quality of medicines: a systematic review
Source: BMJ Glob Health. 2018 Aug 29;3(4):e000725. doi: 10.1136/bmjgh-2018-000725 (PMC6135480; doi:10.1136/bmjgh-2018-000725)
Supplement: Supplementary data [file bmjgh-2018-000725supp007.pdf]

Supplementary file 7. Studies and devices (devices with a *) excluded from the review because of the investigation of performances of chemometrics techniques rather than device(s) performances

Name of the device(s) (developer)	Reference
Antaris II FT-NIR Analyzer (ThermoScientific)*/RXN1 (Kaiser Optical)	[1]
Luminar 3070 (Brimrose Corp)*	[2]
ARCspectro FT-NIR (ThermoScientific)*	[3]
Antaris One (ThermoScientific)*/MPA spectrophotometer (Bruker Optics)/FTLA2000-PH60 (ABB-Bomem)*	[4]
Nicolet iS10 FT-IR (ThermoScientific)	[5] [6]
Antaris II FT-NIR Analyzer (ThermoScientific)*	[7]
RXN1 (Kaiser)	[8]
MATRIX-F (Bruker Optik GmbH)	[9]
i-Raman (B & W Tek)	[10]
MATRIX-F (Bruker Optik GmbH)/EQUINOX55 (Bruker Optic GmbH)*	[11]
SCiO (Consumer Physics)/MicroPhazir RX (ThermoScientific)	[13]
MPA spectrophotometer (Bruker Optics)	[14] [16] [23] [24] [29]
EZ-Raman I (Enwave Optronics Inc.)	[15]
Antaris II FT-NIR Analyzer (ThermoScientific)*/Nicolet 6700 FT-IR spectrometer (ThermoScientific)*/Confocal-Micro-Raman spectrometer HR 800 (LabRAM Raman series, Jobin Yvon)*	[17]
MiniRaman II (B & W Tek)	[12] [18] [19]
Sapphire NIR-CI 2450 (Malvern)*	[20]
Alpha Fourier transform infrared - unnamed (Bruker Optics)	[21]
MPA spectrophotometer (Bruker Optics)/RamanStation 400F (Perkin)*	[22]
MicroNIR Pro 1700 (Viavi Solutions Inc)	[25] [27]
RamSpec (Bayspec)*/Miniature Agility*/Xantus-2/Xantus-1/Xantus-Mini(Rigaku)*	[26]
Truscan (ThermoScientific)	[28]

1. Been F, Roggo Y, Degardin K, Esseiva P. Providing a forensic approach for fighting against counterfeit medicines. Spectra Anal. 2014;43(298):51–62.
2. Cogdill RP, Herkert T, C.A. A. Synthetic calibration for efficient method development: Analysis of tablet API concentration by near-infrared spectroscopy. Vol. 2, Journal of Pharmaceutical Innovation. 2007. p. 93–105.
3. Costa FSL, Pedroza RHP, Porto DL, Amorim MVP, Lima KMG. Multivariate control charts for simultaneous quality monitoring of isoniazid and rifampicin in a pharmaceutical formulation using a portable near infrared spectrometer. J Braz Chem Soc. 2015;26(1):64–73.
4. A. C, J.-S. S, L. C. Quality control of multi-component, intact pharmaceutical tablets with three different near-infrared apparatuses. Vol. 13, Pharmaceutical Development and Technology. N. Abatzoglou, Department of Chemical Engineering, Universite de Sherbrooke, 2500 boul. de l'Universite, Sherbrooke, QC J1K 2R1, Canada. E-mail: Nicolas.Abatzoglou@USherbrooke.ca: Informa Healthcare (69-77 Paul Street, London EC2A 4LQ, United Kingdom); 2008. p. 333–43.
5. Deconinck E, Bothy JL, Barhdadia S, Courselle P. Discriminating nicotine and non-nicotine containing e-liquids using infrared spectroscopy. Vol. 120, Journal of Pharmaceutical and Biomedical Analysis. p. 333–41.
6. Deconinck E, Cauwenbergh T, Bothy JL, Custers D, Courselle P, De Beer JO. Detection of sibutramine in adulterated dietary supplements using attenuated total reflectance-infrared spectroscopy. J Pharm Biomed Anal. 2014;100:279–83.
7. Degardin K, Guillemain A, Guerreiro NV, Roggo Y. Near infrared spectroscopy for counterfeit detection using a large database of pharmaceutical tablets. J Pharm Biomed Anal. 2016;128:89–97.

8. Dégardin K, Roggo Y, Been F, Margot P. Detection and chemical profiling of medicine counterfeits by Raman spectroscopy and chemometrics. Anal Chim Acta. 2011;705(1–2):334–41.
9. Feng Y-C, Yang X-L, Yang Z-H, Hu C-Q. Monitoring the quality of drugs in circulation using rapid NIR spectral comparison methods. J Chinese Pharm Sci. 2011;20(3):290–6.
10. Lu F, Weng X, Chai Y, Yang Y, Yu Y, Duan G. A novel identification system for counterfeit drugs based on portable Raman spectroscopy. Chemom Intell Lab Syst. 2013;127:63–9.
11. Feng Y-C, Zhang X-B, Hu C-Q. Construction of identification system for non-invasive analysis of macrolides tablets using near infrared diffuse reflectance spectroscopy. J Pharm Biomed Anal. 2010 Jan;51(1):12–7.
12. Gryniewicz-Ruzicka CM, Arzhantsev S, Pelster LN, Westenberger BJ, Buhse LF, Kauffman JF. Multivariate calibration and instrument standardization for the rapid detection of diethylene glycol in glycerin by raman spectroscopy. Vol. 65, Applied Spectroscopy. 2011. p. 334–41.
13. Guillemain A, Degardin K, Roggo Y. Performance of NIR handheld spectrometers for the detection of counterfeit tablets. Talanta. 2017 Apr;165:632–40.
14. Kindenge JM, Tshilombo NK, Chavez P-F, De Bleye C, Sacre P-Y, Mbay JMT, et al. Near infrared spectroscopy, a non-destructive technique to fight against counterfeit medicines. Spectra Anal. 2014;43(298):43–6.
15. Lawson LS, Rodriguez JD. Raman Barcode for Counterfeit Drug Product Detection. Anal Chem (Washington, DC, United States). 2016;88(9):4706–13.
16. Lei Y, Luo Z-Y, Hu C-Q. Rapidly screening counterfeit drugs using near infrared spectroscopy: combining qualitative analysis with quantitative analysis to increase effectiveness. J Near Infrared Spectrosc. 2008;16(3):349–55.
17. Li L, Zang H, Li J, Chen D, Li T, Wang F. Identification of anisodamine tablets by Raman and near-infrared spectroscopy with chemometrics. Spectrochim Acta A Mol Biomol Spectrosc. 2014;127:91–7.
18. Loethen YL, Rodriguez JD. Field-deployable Raman anti-counterfeit screening of Tamiflu capsules. Am J Anal Chem. 2015;6(6):559–68.
19. Loethen YL, Kauffman JF, Buhse LF, Rodriguez JD. Rapid screening of anti-infective drug products for counterfeits using Raman spectral library-based correlation methods. Anal (Cambridge, United Kingdom). 2015;140(21):7225–33.
20. Lopes MB, Wolff J-C, Bioucas-Dias JM, Figueiredo MAT. Near-Infrared Hyperspectral Unmixing Based on a Minimum Volume Criterion for Fast and Accurate Chemometric Characterization of Counterfeit Tablets. Anal Chem (Washington, DC, United States). 2010;82(4):1462–9.
21. Marson BM, De Oliveira Vilhena R, De Souza Madeira CR, Pontes FLD, Piantavini MS, Pontarolo R. Simultaneous quantification of artesunate and mefloquine in fixed-dose combination tablets by multivariate calibration with middle infrared spectroscopy and partial least squares regression. Malar J. 2016;15:109/1-109/8.
22. Mbinze JK, Sacre P-Y, Yemoa A, Mavar Tayey Mbay J, Habyalimana V, Kalenda N, et al. Development, validation and comparison of NIR and Raman methods for the identification and assay of poor-quality oral quinine drops. J Pharm Biomed Anal. 2015;111:21–7.
23. Puchert T, Lochmann D, Menezes JC, Reich G. Near-infrared chemical imaging (NIR-CI) for counterfeit drug identification-A four-stage concept with a novel approach of data processing (Linear Image Signature). J Pharm Biomed Anal. 2010;51(1):138–45.
24. Sakamoto T, Fujimaki Y, Hiyama Y. NIR spectroscopic investigation of two fluoroquinolones, levofloxacin and ofloxacin, and their tablets for qualitative identification of commercial products on the market. Pharmazie. 2008;63(9):628–32.
25. Sun L, Hsiung C, Pederson CG, Zou P, Smith V, von GM, et al. Pharmaceutical Raw Material Identification Using Miniature Near-Infrared (MicroNIR) Spectroscopy and Supervised Pattern Recognition Using Support Vector Machine. Appl Spectrosc. 2016;70(5):816–25.
26. Yang W, Wu HW, Qian J, Chandler L, Lieber C, Dentinger C. Multi-wavelength Excitation Raman Spectrometers and Microscopes for Measurements of Real-world Samples. Vol. 8546, Optics and Photonics for Counterterrorism, Crime Fighting, and Defence Viii.
27. Correia RM, Domingos E, Tosato F, et al. Portable near infrared spectroscopy applied to abuse drugs and medicine analyses. Anal Methods 2018;10:593–603. doi:10.1039/C7AY02998E
28. Kakio T, Hiroshima T, Ikeda Y, et al. Survey to Identify Substandard and Falsified Tablets in Several Asian Countries with Pharmacopeial Quality Control Tests and Principal Component Analysis of Handheld Raman Spectroscopy. Am J Trop Med Hyg 2018.
29. Hattori Y, Seko Y, Peerapattana J, et al. Rapid identification of oral solid dosage forms of counterfeit pharmaceuticals by discrimination using near-infrared spectroscopy. Biomed Mater Eng 2017;29:1–14. doi:http://dx.doi.org/10.3233/BME-171708
