# Supplementary material for: Field detection devices for screening the quality of medicines: a systematic review
Source: BMJ Glob Health. 2018 Aug 29;3(4):e000725. doi: 10.1136/bmjgh-2018-000725 (PMC6135480; doi:10.1136/bmjgh-2018-000725)
Supplement: Supplementary data [file bmjgh-2018-000725supp005.pdf]

Supplementary file 5. Main characteristics of the devices and performance findings in the included publications

Type of technology	Name of the device (Developer)	Specificities	Resolution	Results of laboratory tests	Therapeutic indication (API tested)	Pharmaceuticals destroyed	Tested through containers ?	Plus	Minus	What medicines could potentially be tested based on chemical structure and specificities of the device	Ref
**Raman**	MiniRam II (B&W Tek, superseded by the i-Raman B&W Tek)	SR 250-3050 cm-1 (350-1800 cm-1 (17)); excitation laser 785 nm; max laser output power 275 mW; thermoelectrically cooled 2048 pixel CCD detector	8 cm-1	-	Unspecified	No	Yes	Good size, weight, robustness; Very good exportability of data; Very good reference library size;Very Good QA/QC	Not very easy to use; Long pre-heating time; Can't produce an analysis report; Quality of spectrum not good; Quality of the model not good	Any solid forms, not liquids containing proteins	[1]
	Raman Rxn1 Microprobe (Kaiser optical)	SR 200-1800 cm-1, excitation laser 785 nm, 1.7 mm x 1.3 mm sampling area, step size 100 micrometers	4 cm-1	100% Acc to distinguish falsified from genuine samples when k-NN and SIMCA algorithm used (reference method not stated)	Erectile dysfunction (SIL)	Yes (tablets spilt in two)	No	-	-	-	[2]
	TruScan RM* (Thermo Scientific, formerly Ahura)	SR 250 to 2875 cm-1; excitation laser 785 nm; Laser power ~ 250 mW	8 to 10.5 cm-1	Se[c]=79%[3]; Sp[c]=99%[3]; Se[d]=100%[3];Sp[d]=99%[3]; Reliability over time (n=11): 100%[3]; Acc[j]: 100% for falsified and genuines[4]; Acc[j] 67% for placebos[4]; 35% for Generics[4]; Repeatibility : 100% (20 measurements of the same tablets at the same position)[4]; Reproducibility: 100% (changes in position in-between 10 measurements)[4]; Acc[j] 100% when measurement through glass containers or transparent blisterpacks (only tested on 1 product), 0% when through white blister (only tested on 1 product)[4]; Reproducibility 100% (2 different operators, only 2 products tested)[4]; Discovery mode: compositions of only 3 out of 27 falsified samples correctly determined[4]; Good results compared with the benchtop lab instrument although the handheld spectra were much noisier[4];Could not differenciate between a placebo and its genuine containing 0.6% API[4]; Acc: 75 to 100% for identification of genuine medicines depending on the compound [5]; Sp[g] (for different brands of different API) = 100%[5]; Sp[g] (for different lots of the same product) = 100% for AL, CHL,IBU, =2/3 for ZNS[5]; Sp[g] (for different brands of the same API)=100% for AS, IBU, ZNS, ASA, 0% for SP[5];Sp[g] (for different dosages of the same	Anti-malarials (AMO,AL,ART,AS,DHA,CHL , SP,AL,AS-AMO, AS,SP,CHL,QUI,AL) [9],[3],[5],[10]; Unspecified [6] different formulations produced by La Roche Ltd[4];Lipid modifying agents (ATO)[11]; Antiepileptic (OX)[6]; Anti-tuberculosis[9] (ISO, RIF)[15]; Antibiotics (ERY, CIP,CEF)[9] ,[12], [15]; Anti-platelet (CLOP) [12]; Cardiovascular medicines (VAL, CAND) [12],[8];Anti-histamine medicines (CET,LOR)[12]; Analgesic and anti-inflammatory medicines (IBU,ASA,PARA) [5]; Minerals/electrolytes (ZNS) [5]; Erectile dysfunction (SIL)[7]	No	Yes (glass vial[13], [4], transparent blister pack[4],[10] [14])	"Very good size, weight, robustness[¥]; Very easy to use[¥]; Good exportability of data[¥]; Quite good analysis report[¥];Very good quality of spectra[¥]; Very good reference library size[¥]; Very Good QA/QC[¥]"[1], 21CFR Part 11 Compliance Documentation[9]; More accurate than the Minilab[9]; Less sensitive to environmental interference than the NIR Phazir[9]; Analyse possible through transparent blister pack[14],[4],[10],[13] (ref [6] showed mixed results-original packaging can interfere) and glass vials[4] (tests on limited number of samples); Discovery mode of the instrument enables to determine the sample chemical composition via a database integrated into the device (more than 8000 references): can sometimes identify the composition in falsified medicines[14],[4]; Instrument software avoids the user to have to perform modeling/chemometrics, making the device user-friendly[4]; The laser did not seem to damage the tablets tested after measures were repeated 20 times[4]; Decisions = user-independent[14];	High noise and lower signal intensity and resolution compared to a benchtop unit[12],[15],[4] (but good comparative match/fail results in study [4]); ; Slightly less easy to use than the NIR Phazir[9]; Tablet holder not big enough for too large products[4]; Long time needs to be spent for signature measurements[4]; Scanning through white blisterpacks does not give accurate results[4]: High-powered laser component must be registered with the customs authority in some countries[9]; Non standard adaptor required for data transfer[9]; Risk of FP if the intensity of the Raman signal is overwhelming (especially for fixed-dose combinations such as many anti-TB, ARV)[10]	Raman active formulations; Discovery mode may provide information on the chemical composition of the sample (but does not work if compounds not in the integrated reference database and does not work well if mixture of too many compounds)[4]; SP can't be tested because of fluorescence=spectra solely due to SUD[9],[5]; AS[5],[10] and QUI[5] strongly fluorescent but high fluorescence signal suggests their presence, no fluorescence their absence[10]; Unable to detect Raman active species at low concentrations or if tablet thick coating present[12]; Risk of FP if the intensity of the Raman signal is overwhelming (especially for FDC such as many anti-TB, ARV)[10]	[1], [3], [4], [5], [6], [7], [8], [9]***, [10]***, [11], , [12], [13], [14],[15] , [16], [17], [18]

Type of technology	Name of the device (Developer)	Specificities	Resolution	Results of laboratory tests	Therapeutic indication (API tested)	Pharmaceuticals destroyed	Tested through containers ?	Plus	Minus	What medicines could potentially be tested based on chemical structure and specificities of the device	Ref
				brands of the same API) =0% for ASA and PARA[5];Sp[g] after subjecting the tablets to stress (24h at 70°C and 85% rel humidity)= 100% for AS, CHL, AL and ZNS, 0% for SP[5]Can differentiate medicines from different manufacturers[6]; Potential FN of crushed finished product of ETH if samples are tested against reference spectra from different manufacturers; Laboratory testing by analysts involved in method validation: Acc[k] for identification of the presence/absence of sildenafil using 3 units of Truscan on 117 samples each (same samples): 92.6% (15 instruments errors and 11 user-related errors - improper execution of sample preparation mostly) and 97.4% using Discover mode ; Laboratory, testing by four analysts not involved in method validation, Acc[k] for identification of the presence/absence of sildenafil using 3 units of Truscan on 10 samples (same samples) : 98.3% (2 instrument-relaed errors); 100% using Discover Mode ; Field testing, testing by unknown number of special agents unfamiliar with instrument and procedure : Acc[k] of the presence/absence of sildenafil (n=14) using one unit of the Truscan RM: 91.7% NB: a special sample preparation (extraction, filtration, addition of silver colloid) had to be performed[7]; Peak intensity from the API in a specific spectrum region shown to increase linearly with increase of APIand good repeatability of the p-value using three different lots of authentic products with 10 replicate measurements[8]				Cheap,easily[7] portable,no need for sample preparation,fast,easy to use,no requirement for electric supply, possible to scan through packaging			
	FirstDefender TruScan* (Thermo Scientific)	SR 250-2900 cm−1; excitation laser 785 nm; Working distance: ~16 mm without nose cone; ~5mm with nose cone	7 to 10.5 cm−1	Lower prediction performance for QAN of API when compared to Phazir NIR and TruDefender FT-MIR	Experimental formulation (non-therapeutic) (ASA/AA/CAF)	Yes	Yes (plastic bag)	Laser focus can be positioned into the sample of interest minimising influence from packaging material - successful tests through plastic bags in study	Poor quantitation of API in powders compared with NIR Phazir device and the Raman TruDefender (Thermo)	-	[19]
	MIRA* (Metrohm)	SR 400-2300 cm-1; excitation laser 785 nm;	12 to 14 cm-1	Using the Metrohm software+specific chemometric	Chemotherapy (DOXO, EPI) (solutions)	No	Yes (glass vial)	-	Only quantitative over limited concentration	-	[20]

Type of technology	Name of the device (Developer)	Specificities	Resolution	Results of laboratory tests	Therapeutic indication (API tested)	Pharmaceuticals destroyed	Tested through containers ?	Plus	Minus	What medicines could potentially be tested based on chemical structure and specificities of the device	Ref
		single-mode diode laser - maximum 75 mW on the sample;		approach, the Acc, Se,Sp of discrimination between different concentrations of EPI and DOXO were 100%; QAN: good quantitation prediction with coefficient of determination (R2)= 0.9999, low Se limits the domain of quantification (linearity range 1.26-2.00 mg/ml for DOXO; 0.55-2.00 mg/ml for EPI)					range (low sensitivity for quantitation)		
	NanoRam* (B&W Tek)	SR: 176-2900 cm-1 ; Excitation laser 785 nm; Laser output up to 300 mW; Detector: TE Cooled Linear CCD Array	~9 cm-1 §	For correct identification of API, Se[e]=100%, Sp[e]=96% [21]; high FP rate for samples with high fluorescence:SP[21]; Cannot detect differences between different batches of the same medicine[21];Cannot discriminate between medicines with the same API from different manufacturers[21]	Anti-malarials [21](AS-AMO,AL,QUI,SP,AS-SP); Analgesics[22] (ASA,PARA); Antibiotics[22] (CIP); Anti-platelet [22] (CLOP); Anti-coagulant [22](WAR); Weight loss medicines [22] (ORL); Cardiovascular medicines [22](PRO, SIM), Erectile dysfunction medicine[22] (VAR)	No	Yes (transparent blister packs[21])	Comes with a library of 110 USP standard pharmaceutical materials [21]; Built-in algorithm with the help of libraries provides an instantaneous answer [22]; Easy-to-use [21], [22]; Tested successfully through transparent blister packs except for tablets with thick blue coating[21]	-	Does not work for tablets with thick blue coating[21]	[21],[22]
	EZ Raman M Analyzer* (Enwave optronics)	SR 250-2000 cm-1; excitation laser 785nm; accumulation time 20s	4-6 cm-1 §	-	Chemotherapy (NEL)	Yes	No	-	-	-	[23]
	CBEx (Metrohm Raman)	Spectral range: 400-2300 cm-1; Laser wavelength: 785nm class 3B laser;"point and shoot' adapter;Vial holder	Can be enhanced with SERS adapter and substrate	Spectra independent of time (day to day variability over 3 consecutive days) and calibration; Cannot reliably distinguish between brands of the same API/co-formulated formulations tested (n=8 API/co-formulated formulations); Different strengths of AL brand could not be distinguished; Spectra not API specific for :coformulated coated intact RHZE tablets (poor quality RHZE may not be correctly identified if one two three API are lacking in the medicine), AMOX intact capsules (but powder analysis API-specific), FUR , OXY; Good agreement of scan through translucent capsule of one PARA brand; SERS substrate analysis did not enhance Raman signal of OXY formulations; Degraded products (artificial degradation of genuine sample under different conditions) of AL,	Antimalarials (AL); Antituberculosis (IREP, IR); Antibiotics (AMOX); Analgesics (PARA, PASAC); Maternal health medicines (OXY); Cardiovascular medicine (FUR)	Both destroyed and not destroyed	Yes	Easy-to-install and intuitive software; Easy to transfer spectra and libraries between different instruments using microUSB cable; Fast; Small; Marginal instrument to instrument variablility; Does not require electricity; Four-digit code to lock on the instrument (internal timer can be set to lock out a user after a set time period)	Libraries can't be done directly from the instrument (require external computer); Malfunction observed in the field study; Software and hardware only in English; Instrument does not have internet or Bluetooth capabilities	Samples with low API content relatively to excipient and other APIs may be challenging; Co-formulated samples more challenging if one APIs has a strong signal compared to other	[18]

Type of technology	Name of the device (Developer)	Specificities	Resolution	Results of laboratory tests	Therapeutic indication (API tested)	Pharmaceuticals destroyed	Tested through containers?	Plus	Minus	What medicines could potentially be tested based on chemical structure and specificities of the device	Ref
				AMOX, PARA and RHZE could be identified in some cases as poor quality depending on the level of degradation							
	EZ-Raman-I (TSI, Inc)	Excitation wavelength 785 nm; CCD detector; laser power 400 mW*; tablet holded, vial/cuvette holder		Qualitative correct identification of API in 4 finished products (analysis of intact product, 2 capsules and 2 tablets) using a Raman barcode method; Quantitative predictionc (dissolution of capsules contents and tablets in water; after establishing calibration models using reference standards) of APIs in the four products with % of the label claim being as far as from 0.1% to 12.0% (NB: on average on 5 dosage forms tested for each product predictions results for one acyclovir, one doxycycline and one amoxicillin out of two products were within 3% of HPLC results); Specificities of the quantitative models tested on 6 related APIs (famciclovir, valacyclovir,levofloxacin,erythromycin,cephalexin,penicillin V) showing that the built models are specific of the APIs of interests	Antibiotics (AMOX, DOXY), Antiviral (Acyclovir)	N (for qualitative analysis), Y (for quantitative analysis)	N	Quantitation	-	-	[24]
MIR Fourier Transform	Nicolet iS 10 (Thermo Scientific)	SR 400 - 4000 cm-1	> 0.4 cm-1 §		Unspecified	Yes	No	Quite Good pre-heating time¥; Easy to use: Quite Good¥; Very Good exportability of data; Good analysis report; Good quality of spectra; Very good reference library size; Good quality of the model; Quite Good cost; Very Good QA/QC ¥”	Not Good size, weight, robustness¥	Solid forms and liquids containing proteins¥	[1]
	MLp (A2 technologies)	Not specified	Not specified	-	Unspecified	Unspecified	Unspecified	Very good size, weight, robustness; Very Good pre-heating time; Easy to use: Good; Very Good exportability of data; Good analysis report; Good quality of spectra; Very good reference library size; Quite Good quality of the model; Very Good cost; Good QA/QC ¥	None specified¥	Solid forms and liquids containing proteins ¥	[1]
	Exoscan*(A2 technologies - now Agilent technologies;	SR 650 - 4000 cm-1 §	4 cm-1 §	-	Unspecified	Unspecified	Unspecified	Good size, weight, robustness; Very Good pre-heating time; Easy to use: Good; Very Good exportability of data;	Can't produce an analysis report¥	Solid forms and liquids containing proteins ¥	[1]

Type of technology	Name of the device (Developer)	Specificities	Resolution	Results of laboratory tests	Therapeutic indication (API tested)	Pharmaceuticals destroyed	Tested through containers ?	Plus	Minus	What medicines could potentially be tested based on chemical structure and specificities of the device	Ref
	specifications quoted for Exoscan 4100)							Good analysis report ;Good quality of spectra; Very good reference library size; Quite Good quality of the model; Good cost; Good QA/QC¥			
NIR-Fourier Transform	MicroPhazir* (Thermo Scientific)	SR 4167 - 6250 cm-1 (1600-2400 nm); Probe to depth of 100 micron from tablet surface; Light source: Tungsten lamp	8-12 nm	Potential FN of crushed finished product of ethambutol if the samples are tested against reference spectra from different manufacturers[17]	Unspecified Calcium Channel Blockers [25]; Unspecified [13]; Antiepileptic (OX)[6]; Anti-tuberculosis (ETH)[17]; Antibiotic (CEF)	No	Yes (transparent PVC blister[25])	'More pleasing' and 'slightly easier' to use than the TruScan (Ahura)[9]	May require complex specific chemometric approach depending to the aim of the test[25]	-	[6], [13],[17], [25],
	Phazir RX* (Thermo Scientific)	SR 6266–4173 cm−1; InGaAs photodiode detector; Light source: Tungsten lamp	19 cm−1	QAN of powders: prediction performance of the portable instrument is comparable to the benchtop FT-NIR spectrometer when using calibration models	Experimental formulation (blend of ASA/AA/CAF)	No	No	Phazir has shown slightly higher prediction of quantity of API in powders compared to TruScan and TruDefender	-	-	[19]
	Phazir RX* (Polychromix)	SR 4167-6250 cm-1 (1600 – 2400 nm); PbS solid-state detector; Light source: Tungsten lamp	-	-	Unspecified[1]; Anti-malarials [9] (AMO,AL,ART, AS,DHA, CHL, SP); Antibiotics[9] (ERY, CIP); Anti-tuberculosis drugs[9] (ISO, RIF); Erectile dysfunction (SIL citrate, TAD, VAR hydrochloride trihydrate)[26]; calcium channel blockers (AML)[26];Anti-depressant (CIT)[27]; Analgesic (PARA+CAF)[27]; Weight loss (ORL)[27];	No	Variable (unable to penetrate through some packaging - specifics not given[9])	21CFR Part 11 Compliance Documentation; more accurate than Minilab [9]; In-built reference library of different compounds (can be updated online[9]);According to the authors Phazir slightly ergonomically more pleasing and easier to use than the TruScan[9]; no customs-related issues or regulatory restrictions; Data transfer with Phazir with USB-easier than for the TruScan[9]; Very good size, lightweight, robustness; "Very Good pre-heating time¥; Very easy to use¥;Very Good exportability of data¥; Very Good analysis report¥;Very good quality of spectra¥; Very good reference library size¥; Very Good QA/QC¥"[1]	Must control for humidity changes, sample position and sample tests - or perform multiple tests[9]; Ability to penetrate through various packaging variable[9]	-	[1],[9]***,[26]***,[27]
	Luminar 5030* (Brimrose)	SR options: 9090-16666 cm-1(600-1100 nm), 5880 - 11750 cm-1 (850-1700 nm), 5555-11110 cm-1 (900-1800 nm), 4350 - 9090 cm-1 (1100-2300 nm); sampling area 6mm at 40mm sample distance §	2-10 nm §	-	Unspecified	Unspecified	Unspecified	Good size, weight, robustness; Good pre-heating time; Easy to use: Good; Very Good exportability of data; Very good quality of spectra; Quite Good reference library size; Good quality of the model; Good cost; Quite Good QA/QC	Can't produce an analysis report	Solid forms, not liquids containing proteins	[1]
	Target Blend Analyzer (Thermo Scientific)	SR 7400-5550 cm-1 §	3.5 cm-1 §	-	Unspecified	Unspecified	Unspecified	Quite Good size, weight, robustness; Easy to use: Quite Good; Very Good exportability of data; Good analysis report; Good quality of spectra; Very Good reference library size; Very	Long pre-heating time; High cost	Solid forms, not liquids containing proteins	[1]

Type of technology	Name of the device (Developer)	Specificities	Resolution	Results of laboratory tests	Therapeutic indication (API tested)	Pharmaceuticals destroyed	Tested through containers ?	Plus	Minus	What medicines could potentially be tested based on chemical structure and specificities of the device	Ref
								Good quality of the model; Very Good QA/QC			
NIR-Dispersive	MicroNIR 1700* (JDSU) superseded by other MicroNIR (Viavi solution)	SR (950-1650 nm) (NB customized wavelength ranges are possible33); 128 pixel InGaAs photodiode array detector; Sample working distance:0–15 mm from window, 3 mm optimal	Optical: <1.25% of center wavelength; Geometric: 6.25nm per pixel; Pixel size/pitch: 30 µm x 250 µm/50 µm	QAL: 6/6 falsified or illegal generic samples correctly identified [28]; QAL: 2 FP out of 22 raw materials tested [28]; QAN: 0.2% (w/w) of error prediction for quantifying ASA,AA,CAF in blends[28]; Acc of 96% for material conformity, Se[b]=98% and Sp[b]=98.5% for mixtures identification of street drugs, promising reproducibility of direct calibration transfer for new serial numbers/models of the device [29]	QAL: 6/6 falsified or illegal generic samples correctly identified[28]; QAL: 2 FP out of 22 raw materials ID[28]; QAN: 0.6% (w/w) error of prediction for quantifying ASA,AA and CAF blends[28]; Accuracy 96% for material conformity, Se[b]=98% and Sp[b]=98.5% for identification of mixtures in street drugs, promising reproducibility of direct calibration transfer for new serial numbers/models of the device[29]	No	Yes (plastic bags [29])	QAN possible –need more investigation[29]	Requires at least 3 different batches of samples to create reference library [28]	Potentially no limitation	[28],[29]
	RxSpec 700Z (ASD)	SR 4000 - 25640 cm-1 (350 - 2500 nm) §	UNK	-	Unspecified	Unspecified	Unspecified	Good size, weight, robustness; Easy to use: Very Good; Good exportability of data; Quite good quality of spectra; Very Good reference library size; Good quality of the model; Very Good QA/QC	Long pre-heating time; Can't produce an analysis report; High cost	Solid forms, not liquids containing proteins	[1]
	SCiO (Consumer Physics)	Small optical integrating attachment similar to an integrating sphere; Spectral range: 740-1,060 nm	UNK	Se[c] and Sp[c]:100% to distinguish between good quality and falsified AS monotherapy and AL combination; QAN: AMO could not be quantified as the spectra of reference standard was not distinguishable in the spectral signature of therapies containing amodiaquine; Quantitation of AS in one brand (AS-AMO co-formulated in separate tablets) : quantitation of AS within ±14.8% with 95% certainty	Anti-malarials (AL,AS, AS-AMO, AS-SUM-PYR, DHA-PIP)	No	No	Potential to quantify API (needs further investigations), Inexpensive; User-friendly	Requires a spectral library of quality-assured medicines	Quantitation may not be possible with some API such as AMO, although a wider wavelength should improve detection of varying amounts of API	[30]
	D-NIRS	SR 6250 - 10000 cm-1 (1000-1600nm); InGaAs-photodiode detector (640 element array); 10 x 10mm incident light area with focus area of 6 x 6 mm; 55mm path length	1nm wavelength resolution, 0.025mm/pixel spatial resolution	-	Experimental sample tablet ((AA, hydroxypropylmethylcellulose) ([31]); (MS, AA, starch, talc) and (talc, MS, lactose, mannitol) ([32]))	No ([32]), Yes ([31] - but specifically to monitor dissolution)	No	Capable of monitoring dissolution characteristics with high Acc; QAN with high Acc	-	-	[31], [32]
Combined NIR/MIR Fourier Transform	TruDefender FT* (Thermo Scientific)	SR 650-4000 cm−1; Michelson interferometer applies the attenuated total reflection (ATR) technique for sample presentation	4 cm−1	-	Experimental formulation (non-therapeutic) (blend of ASA/AA/CAF)	No	No	-	Slightly lower prediction of quantity of API in powders compared to NIR Phazir RX device (better than Raman FirstDefender) due to imperfect contact between the diamond reflection element and the powder	Liquids, powders and solids	[19]

Type of technology	Name of the device (Developer)	Specificities	Resolution	Results of laboratory tests	Therapeutic indication (API tested)	Pharmaceuticals destroyed	Tested through containers?	Plus	Minus	What medicines could potentially be tested based on chemical structure and specificities of the device	Ref
									sample[19][19][19][19][19][19][19]; accessory needed to optimize contact between the samples and the diamond reflection		
	Cary 630 (Agilent)	SR 600 - 5100 cm -1; Michelson interferometer 25mm, 45° §	4 cm-1	Finished products from 2 manufacturers can be differentiated, but different lots within the same manufacturers cannot be distinguished from each other[6]; Rapid detection of finished products containing no APIs[17]	Antiepileptic (OX)[6]; Anti-tuberculosis (ETH)[17]; Antibiotic (CEF)[17]	Yes	No	More effective than Raman/NIR for detecting counterfeits[17]; Possible rapid detection of samples that contain no APIs[17]; Identification of APIs using specific fingerprints in finished products[17]	-	-	[6],[17]
	FT/IR-4100 (JASCO Inc, Tokyo, Japan)	SR 650-4000 cm-1; ATR unit (DuraSampIIR II, Smiths Detection Group, UK) attached	4 cm-1	Able to distinguish between different formulations with same API (i.e. generic versions of branded genuine product)	Lipid-modifying drugs (ATO)	Yes	No	-	-	Crushed tablets	[11]
**Reflectance colour measurement**	X-rite eye-one* (Regensdorf)	SR 13700-26315 cm-1 (380 nm to 730 nm)	10 nm	Analysis of tablets: 15% FP; analysis of packaging: 25% FP(HPLC ref)	Erectile dysfunction (SIL, VAR, TAD)	No	No	Capable of imaging secondary packaging (i.e. reflectance of carton box measured)	Requires updated reference library of spectra; Significant operator variability (esp with tablets); Ambient light interference if the scanner does not adhere properly = may result in low Acc with convex tablet (i.e Cialis)	Any if reference spectra available	[33]
**Low-cost laser absorption/fluorescence**	Counterfeit Drug Indicator-CoDI* (Michael D. Green, CDC)	405 nm laser, detection via a Cadmium sulfide photoresistor coupled to a voltmeter; signal measured with and without red filter over photoresistor	N/A	-	Antimalarial (AL,CHL, SP)	No	No	Cheap, portable, battery-powered, Smartphone image analysis application could provide rapid real-time color comparisons of samples	Should be used in combination to other devices: proposed in a three-tiered strategy		[34]
**Mass spectrometry**	Mini 10 mass spectrometer coupled to ESI and DESI*	Mass range > m/z 500	1 AMU	-	Drugs of abuse (experimental formulation) (blend of methAMPH/COC/heroin), amines (dibutylamine, TRIbutylamine); peptides (non-therapeutic) (bradykinin, synthetic peptide)	Yes	No	DESI allows in-situ sampling	Ionisation method still not miniaturized; rudimentary atmospheric interface	Drugs of abuse, peptides, possibility to extend to small proteins	[35]***
	QDa single quadrupole (Waters) (Single quadrupole (with DART ionization))	Mass range 30-800 m/z, capillary bias 800V, 33V cone voltage, 150C source temperature (for this experiment)	~1 AMU	Low abundance species in samples were not detected (e.g. CLM in falsified samples)	Anti-malarials (AL)	Yes	No	Lower relative energy requirement to other standard MS units	Few settings in voltage and frequency resulting in bias for the collection of ions in to the analyzer; Not user-friendly; Mass accuracy does not allow for confident identification of new compounds; Needs electricity, possibly with a generator; Requires additional gas for testing	Samples that can be ionized	[36]

Type of technology	Name of the device (Developer)	Specificities	Resolution	Results of laboratory tests	Therapeutic indication (API tested)	Pharmaceuticals destroyed	Tested through containers?	Plus	Minus	What medicines could potentially be tested based on chemical structure and specificities of the device	Ref
									using solid phase DART ambient ionization		
**Nuclear quadrupole resonance (NQR)**	Prototype	23 turns on solenoid; 21 micro-H; Q (quality factor) at 3.03 MHz = 76; 3.033 MHz 14N NQR line of AMP and 2.564 MHz 14N NQR line of PARA used; 'pulsed spin locking' data acquisition sequence (pulse width 60 microseconds; pulse separation 422 microseconds (AMP), 1.15 ms (PARA))	N/A	Successful QAN of blister pack of PARA using fixed-pitch RF coil	Analgesic (PARA); antibiotic (AMP capsules)	No	Yes	Accurate in QAN (compared to HPLC)-needs more investigation	Inspection times longer if quadrupolar isotope in low abundance; Requires reference library of spectra	Any containing relatively high abundance of isotope with nuclear spin I > 1/2	[37]
**Paper-based devices**	Paper test card** (prototype)	12-lane cards ([38], [39])	N/A	Se[g] 90-100% for pure API and excipients (drug-dependent[38]; poor Se[g] for ETH in combination with RIF; 73% for RIF cut with potato starch); lower Se[g] (e.g. 40% for PYR in SP combination) when only small quantity of API[39]); Sp[g] 88-100%[38] (drug-dependent; poor SP[g] (83%) for ETH in combination with RIF[38])	Antibiotics(AMP[38], AMOX[38]); Anti-tuberculosis drugs[38](RIF, ETH, ISO, PYR) ; common adulterants[38], [39] (PARA, talc, baking soda, chalk, QUI, and DIP); Anti-malarials[39](CHL, DOXY, QUI, SUD, PYR); ASA[39]; CAC	Yes	No	Inexpensive; no consumables required;able to identify wide range of compounds	Interpreting result can be challenging; requires authentic samples	Specifically designed for drugs tested; further development needed to increase range of available APIs	[38],[39]
	aPAD**	Paper test card	N/A	Good differentiation of levels of AMOX that differed by 0.15 mg/ml.[40] ; Semi-QAN analysis[c]: AMOX error: 13.0%, systematic bias:11.2%,Inter-device precision (one sample tested 5 times): 0%, AMPI error: 4.7%, systematic bias:3.2%,Inter-device precision (one sample tested 5 times): 2.2%, Inter-reading precision (three analysts): 0.6%; QAL analysis Pass/Fail (identification of samples below or above the USP 90.0% limit of stated API): AMOX Sec:73.2%, Spc:100.0%, AMPI Sec:80.0%, Spc:100.0% NB: authors suggest that artificially degraded samples (thermally stressed) used in the study may have anomalously led to wrong results[41]	Antibiotics (AMOX[40], AMPI)[41]	Yes	No	Inexpensive; semi-QAN	Limited to beta-lactam antibiotics; Destructive, Sample preparation, Slow analysis, Require lab equipment (e.g. balance),non-specific (cannot differentiate beta-lactams from one another)	Beta-lactam antibiotics	[40],[41]

Type of technology	Name of the device (Developer)	Specificities	Resolution	Results of laboratory tests	Therapeutic indication (API tested)	Pharmaceuticals destroyed	Tested through containers ?	Plus	Minus	What medicines could potentially be tested based on chemical structure and specificities of the device	Ref
	Paper-based microfluidic strip - unnamed**	pH 4 required for AS; Calibrated colour chart guide printed onto device for quantitation; "ColorAssist" iPhone application to improve Acc of quantitation	N/A	Successful identificationg of AS in 3 tablets; LOD:[1] 0.98 mg/ml	Anti-malarial (AS)	Yes	No	Inexpensive; semi-QAN	Specific to AS	AS only	[42]
**Lateral flow immunoassay dipstick**	Unnamed**	Specific monoclonal antibodies; test line disappears if the concentrations of API are above the LOD	N/A	AS RDT: No cross-reactivity with AS, AMO,PYN,PIP,PRI,PYR,QUI,CHL,LUM at 20,000 ng/ml [43], LOD 40-50 microg/ml [44] and 1000-2000 ng/ml [43] ; LOD 4-8 microg/ml for ART [44]	Antimalarials (AR derivatives: AS, DHA, ART)	Yes	No	Simple sample preparation; Fast ; Inexpensive; Minimal training: RDT known by healthcare providers (e.g for malaria diagnosis); 95% alcohol as solvent -non-toxic for AS and ART; no interference from partner drugs in assays; highly specific	May be difficult to obtain solvents in remote settings; Shelf life under tropical conditions not yet validated; Currently limited to a few API; Single use; Several tests must be run if failed sample [44]	Highly specific to AR derivatives, but antibody method may be possible for other drugs	[43],[44],[45]
**Dissolution microfluidics with luminescence detection**	PharmaChk beta 1.1	Staggered herringbone chip design; Luminescence measured with photodiode	Not specified	Relative SD < 5%; <4% difference in API quantity compared to HPLC reference standard	Anti-malarial (Lever, Camosunate, Glunate - brand names containing AR)	Yes	No	Can obtain API content and kinetic release profile from dissolving tablet; Cheap; aiming to develop system that could test any drug without extensive reference library	Significant variability in results if wrong solvent used (and solvent choice may be manufacturer-specific); aptamers must be developed for each drug	Currently AR only; hoping to develop system that can test any drug (depending on aptamer availability)	[46]
**TLC, colorimetry, disintegration test**	GPHF-Minilab (Global Pharma Health Fund E.V.)	N/A	N/A	'Limited' Se[47]; 29% of extremely non-compliant samples§b for both content and dissolution correctly identified by the Minilab [48] ; Se[a] for both ID test and content test=79% , Se[a] for ID test only=100% [3]; Sp[a] for ID test only=100% [3]	Anti-malarials [47],[16],[9],[49],[50],[48],[3],[51],[4] (SP, AMO, MEF, AR monotherapies, ACTs, CHL, QUI, DOXY, TET, PRI, DHA-PIP-TRI); Anti-tuberculosis[52],[9] (RIF, ISO); Antibiotics[9],[49],[51](ERY, CIP, AMOX, AMP, SUL-TRI, MET)	Yes	No	Well established within academic literature, 800 Minilabs distributed across 95 countries; TLC can be used for the related substances (degradation products)/impurities testing; Inexpensive; Wide range of API currently covered (n=85 including non anti-infective compounds)	Many consumables; Time consuming; Training ++; Requires dedicated climate controlled location with potable water and electricity; Rough estimate of API content; Reliability of detection depends on the operator's visual acuity and users attention to detail: proficiency testing required[51]	Wide range of APIs currently covered (n=85 for Minilab, mainly anti-infectives but also includes non anti-infective compounds); Potentially no limitation	,[3],[9]***,[16],[47],[48][49]***,[50],[51]***,[52],[53]
**Refractometry**	AR200 digital refractometer* (Leica Microsystems)	Light source: 589nm LED ; refractive index range: 1.3300 - 1.5600 §	Acc +/- 0.0001 nD, +/-0.1% solids, +/-0.2% temperature §	Se[c]: AS: 86%; CHL:83% (250mg tablet); CHL:100% (322.5 mg inj); QUI: 98%; SUL: 97% ; Sp[c]: AS: 87%; CHL:73% (250mg tablet); CHL:86% (322.5 mg inj); QUI: 56%; SUL: 64% (NB: for CHL tab, a adjustment factor for interferences had to be applied)	Antimalarials (AS, CHL,QUI,SP)	Yes- except injection (choroquine)	No	Using refractometry (QAN) and colorimetry (QAL) improves Se and Sp (techniques are complementary)	No QAL	Any soluble drug	[1]***
**Reflectance**	SOC-410 Directional Hemispherical Reflectometer* (Surface Optics Corporation)	Measure at 2 different angles of incidence (20° and 60°) and for six discrete wavelength bands in the 0.9µm to 12µm; SR: SWIR, NIR, MWIR, LWIR	Unspecified	Statistically significant differences in directional reflectance for the 4 types of falsified medicines against the genuine were identified for 4 out of 6 spectral bands tested - small SD indicating high Se and	Erectile dysfunction medicine (SIL)	No	No	Easy to analyse (no need for chemometrics);Sensitive to chemical composition and physical parameters of the surface of the tablets (e.g. difference of compression force during tableting)	Solid oral dosage forms only(although minor modifications could allow the analysis of other forms)	Solid oral dosage forms	[54]

1

Type of technology	Name of the device (Developer)	Specificities	Resolution	Results of laboratory tests	Therapeutic indication (API tested)	Pharmaceuticals destroyed	Tested through containers?	Plus	Minus	What medicines could potentially be tested based on chemical structure and specificities of the device	Ref
				Sp to chemical and physical parameters changes							
	Glossmeter-Unnamed (University of Eastern Finland)	Light source=semiconductor laser	UNK	Data of average gloss obtained consistent with data measured by a 2D glossmeter and the optical profilemeter;NB very limited sample size (n=2)	Antimalarials (AL)	N	N	Fast; Low level of expertise	-	Not liquids	[55]
**Capillary electrophoresis**	Unnamed	Spellman high voltage unit (UM20*4,12V 200 µA) with safety Perspex cage;Miniaturized high voltage- Contactless conductivity detector (C4D built in-house); Flowcell interface in a Plexiglas Block; Screens for monitoring of voltage and current values;rotary selector;Solenoid valves;Gas pressurized buffer container;Function generator in the surface mount technology format;Pick-up amplifier; Rectifier; Low-noise operational amplifiers;Record of the signals with an ADC-20 data acquisition system connected to the USD-port of a computer	~0.5 ppm	Good agreement with quantitation by HPLC	β-agonists (SAL, MTP)	Yes	No	Robust in day-to-day operation; Small sample volumes compared to automated machines; C4D detector gives better separation efficiencies.	Poor tolerance to changing temperature (drift in migration time)	-	[56]
**Ion Mobility Spectrometry**	IONSCAN-LS (Smiths Detection, Danbury)	Desorber T°291C (22, 23); MW range 15-1500 AMU (manufacturer)	0.6 ('resolving power', mass resolution not possible to calculate in IMS due to variation in mobility linked to ion size and shape)	-	Weight loss (SIB[57]); erectile dysfunction (SIL, thiomethisoSI, thioSI, homoSI, methesoSI, VAR, piperidenafil, TAD (as adulterant in herbal supplement for erectile dysfunction) [57]	Yes	No	Ultra-fast quantitative analysis; Sub-nanogram Se; selective; low cost per sample §	-	Solids and liquids	[57]***, [58]
	SABRE 4000*(Smiths Detection, Danbury)	Desorber T° 150-210C in 10C increments; scan time 15s	UNK	-	Stimulant (CAF ([58])), vitamin (vitamin B6 ([58]))	Yes	No	-	-	-	[58]
**Camera system with various LED sources**	CD3/CD3+*(US FDA)	Two charged couple device cameras to capture images and videos (one operates in the UV-Vis; one in the IR spectral region);	Capture of jpeg images at 96 dots per inch resolution (dimensions of image=720x57	Se[c]=100% for analysis based on packaging materials and dosage unit [3], Se[c]=100% for dosage-unit-only analysis[3]; Sp[c]=53% for analysis based on packaging materials and dosage unit [3],	Anti-malarials[3],[59],[34] (AS, AL, AS-AMO), Erectile dysfunction medicines [60] (SIL, TAD), Weight-loss medication [60](ORL)	No	Yes	Screens both packaging and dosage unit (therefore can identify repackaged, degraded drugs); More reliable to conduct side-by-side comparisons with physical	Should be used in combination with other devices such as Raman or FT-IR for better performances [60]	Unlimited	[3], [34],[59] ,[60]

Type of technology	Name of the device (Developer)	Specificities	Resolution	Results of laboratory tests	Therapeutic indication (API tested)	Pharmaceuticals destroyed	Tested through containers?	Plus	Minus	What medicines could potentially be tested based on chemical structure and specificities of the device	Ref
		digital camera can be used and images analysed using smartphone application; Image analysis software has been integrated in a newer version of the device (the 'CD4')[34]	6 pixels at 24-bit color depth (file size approx 75 Kb))	Sp[c]=64% for analysis dosage-unit-only analysis [3]; Se[a]=98.4% (95% CI=93.8-99.7%) [59] ; Sp[a]=100.0 (95% CI=93.8-100.0%) [59]; Inter-user reliability (3 users)= 100% (K=1)[59]				authentic samples than using the library images CDAIL[3]			
**Pressure changes measurement (respirometer system)**	Speedy Breedy (Bactest)	Two chambers	N/A	Against incubator and UV-Vis spectrophotometer: laboratory evaluation results on samples of water for injection only[Ω]: Se 100% under condition A and 93.0% under condition B; Against incubator and UV-Vis spectrophotometer: laboratory evaluation on samples of water for injection only[Ω]: Sp 100% under condition A and B	Sterile water, Maternal health medicine 'OXY), Antimalarial (AS) injections	Yes	N/A	Instrument withstood temperatures between ambient and 40°C during transportation; Small; Light; Easy to transport; Technical support by manufacturers prompt and efficient; Easy-to-download and intuitive software; Hardware and software available in English, Chinese, German, Romanian and Spanish.	Power required (power interruption during the study meant the analysis needed to be repeated);At the time of study, cardboard case making the case not robust for travelling (manufacturer in the process of developing a robust travel case); Vessels only available in 50ml volumes (problems for small volumes samples); Long analysis time and reliable power source required; Only two chambers limit the throughput of the instrument	Liquid samples	[61]

UNK, unknown; Acc, Accuracy; AA, Ascorbic acid; ACT, artemisinin combination therapies; AL,Artemether-Lumefantrine; AML, Amlodipine; AMO, Amodiaquine; AMOX, amoxicillin; AMP, Ampicillin; AMPH, Amphetamine; AR, Artemisinin; ART, Artemether; AS, Artesunate; ASA, Acetylsalicylic Acid; ATO, Atorvastatin; CAC, Calcium carbonate; CAF, Caffeine; CEF, Cefuroxime; CET, Cetirizine; CHL, Chloroquine; CI, Confidence Interval; CIP, Ciprofloxacin; CIT, Citalopram; CLM, Chloramphenicol; CLOP, Clopidogrel; COC, Cocaine; DHA, Dihydroartemisinin; DIA, Diazepam; DIL, Diltiazem; DIP, Diphenhydramine; DOXO, Doxorubicin; DOXY, doxycycline; EPI, Epirubicin; ERY, erythromycin; ETH, Ethambutol; FEN, Fenotropil; FDC, Fixed-Dose Combination; FN, False negative; FP, False positive; FUR, Furosemide; IBU, Ibuprofen; ID, Identification; ISO, Isoniazid; LUM, Lumefantrine; LOR, Loratadine; NEL, Nelabrine; OX, Oxcarbazepine; OXY, Oxycodone; PARA, Paracetamol; PASAC, Paracetamol-acetylsalicylic acid-Caffeine PIP, Piperaquine; PIR, Piracetam; PRI, Primaquine; PRO, Propranolol; PYN, Pyronadine; PYR, Pyrimethamine; MEF, Mefloquine; MET, Metronidazole; MS, Magnesium Stearate; MTP, metoprolol; ORL, Orlistat; OXY, Oxytocin; QAN, Quantitative analysis; QAL, Qualitative analysis; QUI, Quinine; RDT, Rapid Diagnosis Test; RHZE, Rifampicin- Isoniazide-Ethambuthol-Pyrazinamide; RIF, rifampicin; SAL, Salbutamol; Se, sensitivity; SERS, Surface Enhanced Raman Spectroscopy; Sp, specificity; SIB, Sibutramine; SIL, Sildenafil; SIM, Simvastatin; SP, sulfadoxine-pyrimethamine; SR, Spectral Range; SUL, Sulfamethoxazole; SUD, Sulfadoxine; SUM, Sulfamethopyrazine; TAD, Tadalafil; TET, tetracycline; TRI, Trimethoprim; UNK, Unknown; VAL, valsartan; VAR, Vardenafil; WAR, Warfarin; ZNS, Zinc Sulfate

*Handheld, **Handheld- lab-on-a-chip, ***Indicates papers published before 2010

a Against HPLC, Mass spectrometry or Fast Red Dye test assay; b Against Gas chromatography-Mass spectrometry; c Against HPLC API ID and content; d Against HPLC API ID only; e Against TLC (and HPLC for samples failing TLC only), f Against High resolution laboratory grade QTOF mass spectrometer; g No gold standard-tested against known identity of the medicine; h pure reference standards of API (manufacturer supplied) used to benchmark; i One pharmaceutical unit of a medicine was compared to another unit of the same batch; j Unknown reference standard; k Against FTIR or LC-MS

¥The study by Dégardin et al. [1] presents a subjective comparison of multiple devices. Each device feature is described as being 'Very good', 'good', 'quite good' or 'bad' without definition of these NB the final authors' choice of best device per technology is 1:Truscan, 2:Phazir, 3:Mlp

§ Information retrieved from the manufacturer website

Ω the field evaluation was conducted with the aim to determine whether trainees could operate the instrument and whether the instrument could operate in true field-settings;

1. Degardin K, Roggo Y, Margot P. Evaluation of Raman, infrared and near infrared hand held spectrometers for the detection of counterfeit medicines. Spectra Anal. 2010;39(276):46–52.

2. Sacré PY, Deconinck E, Saerens L, De Beer T, Courselle P, Vancauwenberghe R, et al. Detection of counterfeit Viagra by Raman microspectroscopy imaging and multivariate analysis. J Pharm Biomed Anal. 2011;56(2):454–61.

3. Batson JS, Bempong DK, Lukulay PH, Ranieri N, Duane SR, Verbois L. Assessment of the effectiveness of the CD3+ tool to detect counterfeit and substandard anti-malarials. Malar J. 2016;15:119.

4. Degardin K, Guillemain A, Roggo Y. Comprehensive Study of a Handheld Raman Spectrometer for the Analysis of Counterfeits of Solid-Dosage Form Medicines. J Spectrosc. 2017;2017:1–13.

5. Hajjou M, Qin Y, Bradby S, Bempong D, Lukulay P. Assessment of the performance of a handheld Raman device for potential use as a screening tool in evaluating medicines quality. J Pharm Biomed Anal. 2013;74:47–55.

6. Ma B, Wang L. An application of rapid detection technologies in a national regulatory laboratory setting: Differentiating imported and domestic drug products of oxcarbazepine using handheld Raman, near infrared, and portable FTIR analyzers. Am Pharm Rev. 2015;18(2):no pagination.

7. Lanzarotta A, Lorenz L, Batson JS, Flurer C. Development and implementation of a pass/fail field-friendly method for detecting sildenafil in suspect pharmaceutical tablets using a handheld Raman spectrometer and silver colloids. J Pharm Biomed Anal [Internet]. 2017;146:420–5. Available from: http://www.elsevier.com/locate/jpba

8. Kakio T, Yoshida N, Macha S, Moriguchi K, Hiroshima T, Ikeda Y, et al. Classification and Visualization of Physical and Chemical Properties of Falsified Medicines with Handheld Raman Spectroscopy and X-Ray Computed Tomography. Am J Trop Med Hyg. 2017;97(3):684–9.

9. Bate R, Tren R, Hess K, Mooney L, Porter K. Pilot study comparing technologies to test for substandard drugs in field settings. African J Pharm Pharmacol. 2009;3(4):165–70.

10. Ricci C, Nyadong L, Yang F, Fernandez FM, Brown CD, Newton PN, et al. Assessment of hand-held Raman instrumentation for in situ screening for potentially counterfeit artesunate antimalarial tablets by FT-Raman spectroscopy and direct ionization mass spectrometry. Anal Chim Acta. 2008;623(2):178–86.

11. Fukami T, Koide T, Hisada H, Inoue M, Yamamoto Y, Suzuki T, et al. Pharmaceutical evaluation of atorvastatin calcium tablets available on the Internet: A preliminary investigation of substandard medicines in Japan. J Drug Deliv Sci Technol. 2016;31:35–40.

12. Assi S, Watt R, Moffat T. Comparison of laboratory and handheld Raman instruments for the identification of counterfeit medicines. Spectroscopy. 2011;(Suppl.):36,38-44,46-47.

13. Kalyanaraman R, Dobler G, Ribick M. Portable spectrometers for pharmaceutical counterfeit detection. Am Pharm Rev. 2010;13(3):38–45.

14. Kalyanaraman R, Ribick M, Dobler G. Portable Raman Spectroscopy for Pharmaceutical Counterfeit Detection. Eur Pharm Rev. 2012;17(5):11–5.

15. Luczak A, Kalyanaraman R. Portable and benchtop Raman technologies for product authentication and counterfeit detection. Am Pharm Rev. 2014;17(6):76–80.

16. Bate R, Hess K. Anti-malarial drug quality in Lagos and Accra - A comparison of various quality assessments. Vol. 9, Malaria Journal. 2010. p. no pagination.

17. Ma B, Huong LTT, Liu Y, Kamel MM, Zhao E. Rapid detection of counterfeit drugs of ethambutol hydrochloride and cefuroxime axetil using handheld raman, near infrared and portable FTIR technologies. Am Pharm Rev. 2014;17(5):1–13.

18. United States Pharmacopoeial Convention. USP Technology Review: CBEx. [cited 2018 May 8]; Available from: http://www.usp.org/sites/default/files/usp/document/our-work/global-public-health/tr-report-cbex.pdf

19. Sorak D, Herberholz L, Iwascek S, Altinpinar S, Pfeifer F, Siesler HW. New Developments and Applications of Handheld Raman, Mid-Infrared, and Near-Infrared Spectrometers. Appl Spectrosc Rev. 2012 Feb;47:83–115.

20. Le LMM, Tfayli A, Zhou J, Prognon P, Baillet-Guffroy A, Caudron E. Discrimination and quantification of two isomeric antineoplastic drugs by rapid and non-invasive analytical control using a handheld Raman spectrometer. Talanta. 2016;161:320–4.

21. Visser BJ, de Vries SG, Bache EB, Meerveld-Gerrits J, Kroon D, Boersma J, et al. The diagnostic accuracy of the hand-held Raman spectrometer for the identification of anti-malarial drugs. Malar J. 2016;15:160/1-160/12.

22. Assi S. Investigating the quality of medicines using handheld Raman spectroscopy. Eur Pharm Rev. 2014;19(5):56–60.

23. Corrigan DK, Salton NA, Preston C, Piletsky S. Towards the development of a rapid, portable, surface enhanced Raman spectroscopy based cleaning verification system for the drug nelarabine. J Pharm Pharmacol. 2010;62:1195–200.

24. Tondepu C, Toth R, Navin C V, Lawson LS, Rodriguez JD. Screening of unapproved drugs using portable Raman spectroscopy. Anal Chim Acta. 2017;973:75–81.

25. Zontov YV, Balyklova KS, Titova AV, Rodionova OY, Pomerantsev A. Chemometric aided NIR portable instrument for rapid assessment of medicine quality. J Pharm Biomed Anal. 2016;131:87–93.

26. Polli JE, Hoag SW, Flank S. Near-infrared spectrophotometric comparison of authentic and suspect pharmaceuticals. Pharm Technol. 2009;33(8):46–52.

27. Obeidat SM, Al-Tayyem B. Spectroscopic and chemometric analysis of illegally manufactured formulations of selected medicines. Orient J Chem. 2012;28(2):795–801.

28. Alcala M, Blanco M, Moyano D, Broad NW, O'Brien N, Friedrich D, et al. Qualitative and quantitative pharmaceutical analysis with a novel hand-held miniature near infrared spectrometer. J Near Infrared Spectrosc. 2013;21(6):445–57.

29. Pederson CG, Friedrich DM, Hsiung C, von Gunten M, O'Brien NA, Ramaker H-J, et al. Pocket-size near-infrared spectrometer for narcotic materials identification. In: Next-Generation Spectroscopic Technologies VII, Proceedings of SPIE Vol 9101, 91010O. 2014. p. 91010O–1–

11.

30. Wilson BK, Kaur H, Allan EL, Lozama A, Bell D. A New Handheld Device for the Detection of Falsified Medicines: Demonstration on Falsified Artemisinin-Based Therapies from the Field. Am J Trop Med Hyg. 2017 Feb;96(5):1117–23.

31. Ishikawa D, Murayama K, Awa K, Genkawa T, Komiyama M, Kazarian SG, et al. Application of a newly developed portable NIR imaging device to monitor the dissolution process of tablets. Anal Bioanal Chem. 2013;405(29):9401–9.

32. Ishikawa D, Murayama K, Genkawa T, Awa K, Komiyama M, Ozaki Y. Development of a compact near infrared imaging device with high-speed and portability for pharmaceutical process monitoring. NIR news. 2012;23(8):14–7.

33. Rodomonte AL, Gaudiano MC, Antoniella E, Lucente D, Crusco V, Bartolomei M, et al. Counterfeit drugs detection by measurement of tablets and secondary packaging colour. J Pharm Biomed Anal. 2010;53(2):215–20.

34. Green MD, Hostetler DM, Nettey H, Swamidoss I, Ranieri N, Newton PN. Integration of novel low-cost colorimetric, laser photometric, and visual fluorescent techniques for rapid identification of falsified medicines in resource-poor areas: application to artemether-lumefantrine. Am J Trop Med Hyg. 2015 Jun;92(Suppl 6):8–16.

35. Keil A, Talaty N, Janfelt C, Noll RJ, Gao L, Ouyang Z, et al. Ambient mass spectrometry with a handheld mass spectrometer at high pressure. Anal Chem. 2007;79(20):7734–9.

36. Bernier MC, Li F, Musselman B, Newton PN, Fernandez FM. Fingerprinting of falsified artemisinin combination therapies via direct analysis in real time coupled to a compact single quadrupole mass spectrometer. Anal Methods. 2016;8(36):6616–24.

37. Barras J, Katsura S, Sato-Akaba H, Itozaki H, Kyriakidou G, Rowe MD, et al. Variable-pitch rectangular cross-section radiofrequency coils for the nitrogen-14 nuclear quadrupole resonance investigation of sealed medicines packets. Anal Chem. 2012;84(21):8970–2.

38. Weaver AA, Reiser H, Barstis T, Benvenuti M, Ghosh D, Hunckler M, et al. Paper Analytical Devices for Fast Field Screening of Beta Lactam Antibiotics and Antituberculosis Pharmaceuticals. Anal Chem. 2013;85(13):6453–60.

39. Weaver AA, Lieberman M. Paper test cards for presumptive testing of very low quality antimalarial medications. Am J Trop Med Hyg. 2015;92(Suppl 6):17–23.

40. Myers NM, Kernisan EN, Lieberman M. Lab on Paper: Iodometric Titration on a Printed Card. Anal Chem. 2015;87(7):3764–70.

41. Myers N. Lab on a paper: adapting quantitative chemical techniques for use in low resource areas [Internet]. 2017 [cited 2018 May 9]. Available from: https://curate.nd.edu/downloads/und:m900ns08m33

42. Koesdjojo MT, Wu Y, Boonloed A, Dunfield EM, Remcho VT. Low-cost, high-speed identification of counterfeit antimalarial drugs on paper. Talanta. 2014;130:122–7.

43. Guo S, Zhang W, He L, Tan G, Min M, Kyaw MP, et al. Rapid evaluation of artesunate quality with a specific monoclonal antibody-based lateral flow dipstick. Anal Bioanal Chem. 2016 Sep 12;408:6003–8.

44. Guo S, Wang B, He L, Tisch DJ, Kazura J, Mharakurwa S, et al. Pilot testing of dipsticks as point-of-care assays for rapid diagnosis of poor-quality artemisinin drugs in endemic settings. Trop Med Health. 2016;44:15.

45. He L, Nan T, Cui Y, Guo S, Zhang W, Zhang R, et al. Development of a colloidal gold-based lateral flow dipstick immunoassay for rapid qualitative and semi-quantitative analysis of artesunate and dihydroartemisinin. Malar J. 2014;13(1):127.

46. Desai D. PharmaChk: Robust device for counterfeit and substandard medicines screening in developing regions. [Internet]. 2014. Available from: https://open.bu.edu/handle/2144/12087

47. Visser BJ, Meerveld-Gerrits J, Kroon D, Mougoula J, Vingerling R, Bache E, et al. Assessing the quality of anti-malarial drugs from Gabonese pharmacies using the MiniLab: A field study. Malar J. 2015;14(1):no pagination.

48. World Health Organization. Survey of the quality of Selected antimalarial medicines circulating in Six countries of Sub-Saharan africa [Internet]. Geneva, Switzerland; 2011 [cited 2017 Aug 28]. Available from: http://apps.who.int/medicinedocs/en/d/Js17835en/

49. Risha PG, Msuya Z, Clark M, Johnson K, Ndomondo-Sigonda M, Layloff T. The use of Minilabs to improve the testing capacity of regulatory authorities in resource limited settings: Tanzanian experience. Health Policy. 2008;87(2):217–22.

50. Pribluda VS, Barojas A, Anez A, Lopez CG, Figueroa R, Herrera R, et al. Implementation of basic quality control tests for malaria medicines in Amazon Basin countries: results for the 2005-2010 period. Malar J. 2012;11:202.

51. Risha P, Msuya Z, Ndomondo-Sigonda M, Layloff T. Proficiency testing as a tool to assess the performance of visual TLC quantitation estimates. J AOAC Int. 2006;89(5):1300–4.

52. Bate R, Jensen P, Hess K, Mooney L, Milligan J, R. B, et al. Substandard and falsified anti-tuberculosis drugs: a preliminary field analysis. Int J Tuberc Lung Dis. 2013;17(3):308–11.

53. Pan H, Ba-Thein W. Diagnostic Accuracy of Global Pharma Health Fund Minilab ™ in Assessing Pharmacopoeial Quality of Antimicrobials. Am J Trop Med Hyg. 2017;tpmd170289.

54. Wilczyński S, Koprowski R, Błońska-Fajfrowska B. Directional reflectance analysis for identifying counterfeit drugs: Preliminary study. J Pharm Biomed Anal. 2016;124:341–6.

55. Bawuah P, Paakkonen P, Peiponen K-E. Gloss measurement in detection of surface quality of pharmaceutical tablets: a case study of screening of genuine and counterfeit antimalaria tablets. Acad-Rapid Publ. 2017;13:18.

56. Nguyen TAH, Pham TNM, Doan TT, Ta TT, Saiz J, Nguyen TQH, et al. Simple semi-automated portable capillary electrophoresis instrument with contactless conductivity detection for the determination of beta-agonists in pharmaceutical and pig-feed samples. J Chromatogr A. 2014;1360:305–11.

57. Gryniewicz CM, Reepmeyer JC, Kauffman JF, Buhse LF. Detection of undeclared erectile dysfunction drugs and analogues in dietary supplements by ion mobility spectrometry. J Pharm Biomed Anal. 2009;49(3):601–6.

58. Dunn JD, Gryniewicz-Ruzicka CM, Kauffman JF, Westenberger BJ, Buhse LF. Using a portable ion mobility spectrometer to screen dietary supplements for sibutramine. J Pharm Biomed Anal. 2011;54:469–74.

59. Ranieri N, Tabernero P, Green MD, Verbois L, Herrington J, Sampson E, et al. Evaluation of a new handheld instrument for the detection of counterfeit artesunate by visual fluorescence comparison. Am J Trop Med Hyg. 2014;91(5):920–4.

60. Lanzarotta A, Ranieri N, Albright D, Witkowski M, Batson J. Analysis of Counterfeit FDA-Regulated Products at the Forensic Chemistry Center: Rapid Visual and Chemical Screening Procedures Inside and Outside of the Laboratory. Am Pharm Rev. 2015;18(May):no pagination.

61. United States Pharmacopoeial Convention. USP Technology Review: Speedy Breedy. [cited 2018 May 8]; Available from: http://www.usp.org/sites/default/files/usp/document/our-work/global-public-health/technology-review-report-speedy-breedy.pdf
