# Supplementary material for: Field detection devices for screening the quality of medicines: a systematic review
Source: BMJ Glob Health. 2018 Aug 29;3(4):e000725. doi: 10.1136/bmjgh-2018-000725 (PMC6135480; doi:10.1136/bmjgh-2018-000725)
Supplement: Supplementary data [file bmjgh-2018-000725supp006.pdf]

Supplementary file 6. Practical characteristics of the devices for use in the field

	Name of the device (Developer)	Weight (kg)/Dimensions (cm)	Calibration and performance maintenance	Operating temperature range	Warm-up time	Tolerance to operational environment changes	Electricity requirements	Consumables requirements	End-user skill level required	Measurement time per sample	Additional equipment required	Other information	Ref
Raman	MiniRam II (B&W Tek)	2.9/25.7x 21.1x11.4 §	UNK	UNK	UNK	UNK	Battery-powered (3 hrs life) §	None	UNK	<5 min	UNK		[1]
	Raman Rxn1 Microprobe (Kaiser optical)	28kg/ 58 x 45 x 20 §	UNK	20-25°C §	20 min §	20-80% humidity §	110-240V, <200W §	None	UNK	30s	Software, computer	One sample per run	[2]
	TruScan RM* (Thermo Scientific, formerly Ahura)	0.9/20.8x10.7x4.3 §	UNK	-20-40°C §	UNK	UNK	Battery-powered (Rechargeable internal lithium ion battery > 3 hrs life) or Mains-powered (100-240 V AC 50/60 Hz)	None	+	Maximum 2 min (+ 5-30 min to create the reference library)[3],[4],[5],[6]	Reference library software; Vial holder, tablet holder	One sample per run	[3],[4] [5], [6],[7] ,[8], , [9],[10], [11]
	TruScan* (Ahura)	<1.8/30x15x7.6 §	Fast and easy calibration[1]¥;[12]; requires reference rods (provided by Ahura)[13]	-20-40°C	Good[1]¥	Less sensitive to external factors than Phazir[12]	Battery-powered (Internal lithium ion battery, >5 hrs life at 25 °C) or Mains-powered (100-240 V AC 50/60 Hz §	None	+	30s to 5 min [12],[14]	Non standard adaptor for data transfer	Safety precautions: high-power laser component[12]- One sample per run	[1], [12]*, [13],[14],[15] , ,[16]*
	FirstDefender TruScan* (Thermo Scientific)	0.8/19.3x10.7x4.4	UNK	-20-50 °C §	UNK	UNK	Battery-powered (lithium ion battery or 123a batteries; > 4 hrs life or Mains-powered (DC Wall Adapter, 12 V 1.25 A) §	None	UNK	UNK	UNK	One sample per run	[17]
	MIRA* (Metrohm)	0.54/12.5×8.5×3.9 §	UNK	-20-40 °C	UNK	UNK	Battery-powered §	None	UNK	< 5 min	UNK	One sample per run	[18]
	NanoRam* (B&W Tek)	1.2kg/22x10x5 §	Calibration following the developer procedures, valid for one year[19]	-20-40°C §	UNK	UNK	Battery-powered (Li-ion, >5 hrs life) or Mains-powered (AC adapter: Output DC 12V, 2A Minimum) §	None	+	15 s (10 for entering data, 5s for scanning the sample) [19]; Reference spectrum created in 3.5 min [19]	Point & Shoot, Vial Holder, Polystyrene Validation Cap, Immersion Probe, Large Bottle Adapter §	One sample per run	[19], [20]
	EZ Raman M Analyzer* (Enwave optronics)	~2.7/10.2x15.9x20.9 §	UNK	10-40°C §	UNK	UNK	Battery-powered-Rechargeable Li battery (5 hrs life) or 90 VAC to 264 VAC §	None	UNK	3 min	PC	One sample per run	[21]
	CBEx (Metrohm Raman)	0.335 /9x7x3.75	Daily calibration (calibration standards provided, 2-years lifetime)	-10-40°C	UNK	Can operate in up to 95% non-condensing humidity (manufacturer). Ambient light can cause instrument response issues; however the referencing function generally alleviates these issues.	2 AA batteries or a micro-USB cable connected to a computer	AA batteries can be used (approx. 3h run in the study before having to change the batteries)	Based on feedback from study participants: a variety of staff with both technical and non-technical backgrounds can become either basic, intermediate or advanced	Approx 2 min (5 sec to do scan); 20 min to develop a library for one sample	PC useful to compare spectra; Standalone devce do not give matches with Match score below 0.85	One sample per run	[22]

	Name of the device (Developer)	Weight (kg)/Dimensions (cm)	Calibration and performance maintenance	Operating temperature range	Warm-up time	Tolerance to operational environment changes	Electricity requirements	Consumables requirements	End-user skill level required	Measurement time per sample	Additional equipment required	Other information	Ref
									users within approx. two weeks of training				
	EZ-Raman-I (TSI, Inc)	11.3/43.2x33.0x17.8	UNK	UNK	UNK	UNK	Rechargeable lithium battery (4 hours operation); 110/220 V DC power supply	None	UNK	10-40s acquisition times in study	-		[23]
**NIR-Dispersive**	MicroNIR1700* (JDSU)	0.06/4.5cm diameterx4.2cm height	UNK- NB:Re-zeroing every 15 min in study[24]	-20-40°C	UNK	UNK	USB-powered (<500 mA at 5V)	None	+ with Onsite Software	< 1 min	PC or Tablet; Polyethylene plastic bag with an X heat sealed onto the bag	Bulb life >40,000 hr - One sample per run; Requires polyethylene bag with an X heat sealed onto the bag when the sample is very small [25]	[24],[25]
	SCiO (Consumer Physics)	Smartphone-sized	UNK	UNK	UNK	UNK	UNK	None	+	Acquisition time per spectrum: 2s	Smartphone		[26]
	D-NIRS	< 2/19.1x9.3x12.0	UNK	UNK	UNK	UNK	Mains-powered	None	UNK	3 min (ref [27])	Computer, software		[28],[27]
	RxSpec 700Z (ASD)	UNK/Briefcase-sized'	Very good¥	UNK	Bad¥	UNK	UNK	None	UNK	UNK	UNK		[1]
**NIR-Fourier Transform**	MicroPhazir* (Thermo Scientific)	1.25/25.4x29.2x15.2 §	UNK	+5-45°C §	UNK	Dust proof,splash proof plastic housing §	Battery-powered (5+ hrs lifetiem, lithium-ion battery pack) or Mains-powered §	None	+	< 5 min	Laptop if more complicated chemometrics approaches are used [29]	One sample per run	[3],[9],[10],[29]
	Phazir RX* (Polychromix)	1.8/25.4x29.2x15.2 §	Very Good[1]¥; Fast and easy[12]	+5-45°C §	Very Good[1]¥	Testing needs to be done in a light controlled environment (results altered if ambient light changed significantly); Sensitive to humidity changes,sample position,sample face for tablets - issues that can be overcome by repeated testing [12]; Dust proof,splash proof plastic housing	Battery-Powered (10 hrs life, quick change battery)	None	+	2-5 s	-	One sample per run	[1],[12]*,[30]*,[31]
	Phazir RX* (Thermo Scientific) newly MicroPhazir?	1.8/25.4x29.2x15.2	UNK	+5-40°C	UNK	UNK	UNK	None	+	UNK	Optional adapter is available that can be attached magnetically to the front of the instrument to optimize sample presentation	One sample per run	[17]
	Luminar 5030* (Brimrose)	UNK	Quite good[1]¥	UNK	Good	UNK	Battery-Powered (2 VDC battery) or Mains-powered (110/220V) §	None	UNK	UNK	PC interface with ethernet connection; Windows-based analytical software for data acquisition §		[1]

	Name of the device (Developer)	Weight (kg)/Dimensions (cm)	Calibration and performance maintenance	Operating temperature range	War m-up time	Tolerance to operational environment changes	Electricity requirements	Consumables requirements	End-user skill level required	Measurement time per sample	Additional equipment required	Other information	Ref
	Target Blend Analyzer (Thermo Scientific)	9.9/20.8x35.4x30.9 §	Very good¥	UNK	Bad¥	UNK	Battery-powered (3.5 hrs life) §	None	UNK	UNK	PC, software §		[1]
MIR Fourier Transform	MLp (A2 technologies)	UNK	Good¥	UNK	Very good¥	UNK	UNK	None	UNK	UNK	UNK		[1]
	Nicolet iS 10 (Thermo Scientific)	33/25x57x55 §	Very good¥	UNK	Quite good¥	Tightly sealed to resist ambient humidity §	Mains-powered (100-240 V, 50/60 Hz) §	None	UNK	UNK	UNK		[1]
	Exoscan*(A2 technologies - now Agilent technologies; specifications quoted for Exoscan 4100)	3.2/17.1x11.9x22.4 §	Good[1]¥ (built-in 'performance validation' tests for user to run)	0-50°C §	Very good[1]¥ (5 min - from manufacturer)	Tolerates up to 95% humidity; packaged in 'weather-resistant enclosure' designed for outdoor use; altitude up to 2000m §	Battery-Powered (up to 4 hrs life) or Mains-powered (110/220 VAC §	None	UNK	UNK	Comes with handheld PC as standard; can be interfaced to laptop §	One sample per run	[1]
Combined NIR/MIR Fourier Transform	TruDefender FT* (Thermo Scientific)	1.3/19.6x11.2x5.3	UNK	-25-40 °C §	UNK	UNK	Battery-powered (rechargeable lithium ion battery or 123a -ie SureFire™- batteries; >4 hrs life or Mains-powered (Wall plug transformer 100-240 VAC 50/60 Hz §	None	UNK	UNK	Crusher accessory for powders (to press the samples against diamond refelection element)[17]	Little maintenance [17]	[17]
	Cary 630 (Agilent)	3.8/16x31x13 §	UNK	UNK	1hour	UNK	Mains-powered (110 – 240 VAC, 60/50 Hz) §	None	UNK	UNK	UNK		[9],[10]
	FT/IR-4100 (JASCO Inc, Tokyo, Japan)	33/446x64.5x29 §	UNK	UNK	UNK	UNK	Mains-powered	None	UNK	UNK	UNK		[5]
Camera system with various LED sources	CD3/CD3+* (US FDA)	0.3/15.2x7.6	UNK	UNK	UNK	UNK	Battery-Powered (3 to 8 hrs life) or Mains-powered	None	+ Accuracy improves with experience	< 1 min	Digital handheld microscope can be used to examine suspect samples at higher magnifications [32]; Library software [33],[32]		[33],[32],[34],[35]
Low-cost laser absorption/fluorescence	Counterfeit Drug Indicator-CoDI* (Michael D. Green, CDC)	UNK	UNK	UNK	None§	UNK	9V alkaline battery§	Aluminium foil§	+	< 1 min	-	-	[34]
Reflectance	SOC-410 Directional Hemispherical Reflectometer* (Surface Optics Corporation)	29.3x22.9x9.4 §	UNK	0-40°C §	UNK	UNK	Battery-powered §	None	+	5 seconds (measurement at one spectral band and one angle)	UNK	-	[36]
	Glossmeter-Unnamed (University of Eastern Finland)	UNK	UNK	UNK	UNK	UNK	Rechargeable battery	None	+	UNK	UNK	Calibration with a commercial black glass gloss standard in the study	[37]

	Name of the device (Developer)	Weight (kg)/Dimensions (cm)	Calibration and performance maintenance	Operating temperature range	Warm-up time	Tolerance to operational environment changes	Electricity requirements	Consumables requirements	End-user skill level required	Measurement time per sample	Additional equipment required	Other information	Ref
**Refractometry**	AR200 digital refractometer* (Leica Microsystems)	0.41/18x9x3.5 §	Simple calibration system built into machine §	10-45°C;Temperature dependent testing but adding blank and reference standard to confirm the integrity of the assay	UNK	Refractive index is temperature-dependent; max tolerated humidity 50-80% (temperature-dependent; tolerated humidity decreases with increasing ambient temperature), pollution degree 2, altitude up to 2000m	Battery-powered (4xAAA batteries) §	+++ glass vials, enteric coating remover solutions, extraction solvents, reference standards; AAA batteries	UNK	UNK	UNK	>3000 tests before replacing batteries	[38]
**Reflectance colour measurement**	X-rite eye-one* (Regensdorf)	0.245/15.5x6.6x6.7 §	UNK- NB: Calibration performed every 10 scans in study	10-35°C	UNK	Ambient light interference if the scanner does not adhere perfectly to the tablet surface;Humidity tolerance 0-80% (non-condensing); Dust and Water resistance IP 65; No influence of temperature (20° and 30°C)	USB-powered §	None	+	Few seconds	Laptop computer with USB port		[39]
**Lateral flow immunoassay dipsticks**	Unnamed**	Few grams	N/A	UNK	None	No significant change in sensitivity when stored at room temperature for 2 weeks [40]; LOD increased after 3 months at 4°C and ambient temperatures for primary made dipsticks targetting all artemisinin derivatives [41] (more investigations are needed for the newer single-API targeted dipsticks)	None	Solvents	+	10 min	Dropper (supplied with plate)	Single use device; Non toxic reagents - Waste management: solvents	[40],[41],[42]
**Paper-based devices**	Paper-based strip – unnamed**	UNK (likely <0.1)/UNK (4 x 8mm filter paper circles on mounted on chip)	N/A	UNK	N/A	Requires controlled pH	None (if smartphone used to read the cards - battery-powered)	Solvents	UNK	5 min (+ 5 min for semi-quantitative analysis using the smartphone application)	Smartphone (to take digital images and measure gray scale intensity-improves quantitative accuracy)	Single use device	[43]
	Paper analytical device**	UNK (likely < 0.05)/Size of a Playing card	N/A	UNK	None	Majority of reactants stable up to 104 days at 37°C (some reactant lanes degrade within 2-7 days of fabrication[44])	None	Water used as solvent	+	10-20 min	None	Single use device; Non toxic reagents – Waste management: All reagents non-toxic	[44], [45]
	aPAD	UNK Size of a playing card	N/A	UNK	None	UNK	None	Solvents, reagents	+ (Successful tests interpretation by n=1 analyst (unknown qualification) unfamiliar to the aPAD [46]	30-60 minutes	Mortar, Pestle, Analytical balance, Pipet;Can use smartphone camera to analyse results (visual inspection adequate)	Single use device	[47],[46]

	Name of the device (Developer)	Weight (kg)/Dimensions (cm)	Calibration and performance maintenance	Operating temperature range	War m-up time	Tolerance to operational environment changes	Electricity requirements	Consumables requirements	End-user skill level required	Measureme nt time per sample	Additional equipment required	Other information	Ref
**TLC, colorimetry,disintegration test**	GPHF-Minilab (Global Pharma Health Fund E.V.)	~50/83x52x29	Performing TLC on reference APIs and reagents	-	None	TLC requires dedicated climate controlled location; Tropics-compatible but avoiding direct sunlight. No special storage area required for the quantities of chemicals supplied.	Electricity required for UV detectors -can be Battery-Powered 9	Reagents;solvents; reference standards;TLC plates;potable water; NB:2 to 5 years shelf-life for authentic secondary reference standards;5 years shelf-life for reagents and solvents in their original packaging	+++ (Medium Lab Skills - Training of at least one-week;proficiency testing highly recommended	30min -1h30 [48],[49]	Lab glassware	No maintenance[49],[7]; 1000 TLC can be run with available solvents/reagents available at purchase; Safety precautions:some toxic/inflammable solvents/reagents - Waste management: must dispose of TLC solvents	[7],[12] ,[15],[48], [49]*, [50], [51],[52], [53]*, [54]
**Dissolution microfluidics with luminescence detection**	PharmaChk beta 1.1	8.2/Pelican briefcase'	Inbuilt calibration; Need new stock solution of reference drug each day	UNK	UNK	UNK	Mains-powered (12V power source from 110/220V	Solvents (Acetylnitrile, NaOH); luminol and hematin porcine probe; Stock solution made with 200-proof ethanol	UNK	5 min	UNK - PC and software should be provided by the company in the kit	Safety precautions:Acetylnitrile solvent is toxic - Waste management: on-board waste container; can run 10-15 samples before emptying	[55]
**Mass spectrometry**	QDa single quadropole (Waters)	29.4/35.3x20.0x75.0	Internal Calibration performed daily	UNK	10min	UNK	Mains powered 110-240V AC 50/60Hz	Solvents; Gas		Few min for both sample preparation and MS introduction	PC, Software; Ionisation source (some can be transportable but usually requires power and gas)	Needs to remain stationary when in operation.	[56]
**Nuclear quadrupole resonance (NQR)**	Prototype	UNK/Carry-on luggage	UNK	UNK- NB:Spectra recorded at room temperature	UNK	UNK	Battery-Powered (12V Lithium battery) §	None	UNK (aiming minimal)	UNK	12V Li battery, PC		[57]
**Ion mobility spectrometry**	SABRE 4000* (Smiths Detection, Danbury)	3.1/36.8 x10.2x11.4	UNK	UNK	10 min §	UNK	Battery-Powered (4hr life) §	Solvents	UNK	< 1 min	Solvent; pipette; centrifuge; scales able to weight ng weights; software	One sample per run	[58]
	IONSCAN-LS (Smiths Detection, Danbury)	42/62x41x88 §	UNK	UNK	UNK	No significant change in ion mobility with humidity (tested at 60% and 90% relative humidity)[58]	Mains-powered (95-265 VAC) §	Solvents;Nitrogen gas	UNK	UNK	Software, nitrogen gas		[58], [59]*
**Capillary electrophoresis**	Unnamed	UNK	UNK	UNK	UNK	If non-thermostated instrument, measurements can be affected by changing temperatures	Battery-powered (Lithium battery pack (14.8V; 6.6Ah) for electrophoretic and fluidic parts+a pair of Li-ion batteries (2.8 Ah each) for the C4D or Mains-powered	Buffer; Solvents; pH adjusting solutions	UNK	UNK	PC	Safety precautions: safety cage needed for high-voltage components	[60]
**Pressure changes measurement (respirometer system)**	Speedy Breedy (Bactest)	2.75/13.3x31 x11.2	UNK	UNK	UNK	The device is not waterproof and unshielded, so above normal electromagnetic interference could result in ineffective tests. The instrument is robust but not ruggedized and has not	Local mains AC power supply or 12V DC (car adapter is available) Voltage: Variable (230V / 50Hz – 120V / 60Hz)	Media vessels, sterile plastic water bottles, sterile syringes	Based on feedback from study participants: a variety of staff with both technical	Can exceed 24 hours (each protocol has a different run time that is bacteria specific)	None	None	[61]

Name of the device (Developer)	Weight (kg)/Dimensions (cm)	Calibration and performance maintenance	Operating temperature range	Warm-up time	Tolerance to operational environment changes	Electricity requirements	Consumables requirements	End-user skill level required	Measurement time per sample	Additional equipment required	Other information	Ref
					been drop tested. However, provided humidity, dust, and vibration changes are not too rapid or severe, the instrument can tolerate fluctuations very well			and non-technical backgrounds can become either basic, intermediate or advanced users within approx. two weeks of training				

LOD, limit of detection; LOQ, limit of quantitation; TLC, Thin-layer chromatography, AL, Artemether-lumefantrine; API, Active Pharmaceutical Ingredient; ASA, acetylsalicylic Acid; HPLC, High Performance Liquid Chromatography,SP, sulfadoxine-pyrimethamine;Se, Sensitivity; Sp, specificity, RDT, Rapid Diagnostic Test, FRTR: Fast Red Dye Reaction; LOD, limit of detection
*Indicates papers published before 2010
¥The study by Dégardin et al. presents a subjective comparison of multiple devices. Each device feature is described as being 'Very good', 'good', 'quite good' or 'bad' without definition of these NB the final authors' choice of best device per technology is 1:TruScan, 2:Phazir, 3:Mlp
+: minimal (<2 hours training); ++ : low (2 hours to 1 day of training); +++: high (>1 day of training)
§ Information retrieved from the manufacturer website or from contacts with manufacturer

1. Degardin K, Roggo Y, Margot P. Evaluation of Raman, infrared and near infrared hand held spectrometers for the detection of counterfeit medicines. Spectra Anal. 2010;39(276):46–52.
2. Sacré PY, Deconinck E, Saerens L, De Beer T, Courselle P, Vancauwenberghe R, et al. Detection of counterfeit Viagra by Raman microspectroscopy imaging and multivariate analysis. J Pharm Biomed Anal. 2011;56(2):454–61.
3. Kalyanaraman R, Dobler G, Ribick M. Portable spectrometers for pharmaceutical counterfeit detection. Am Pharm Rev. 2010;13(3):38–45.
4. Kalyanaraman R, Ribick M, Dobler G. Portable Raman Spectroscopy for Pharmaceutical Counterfeit Detection. Eur Pharm Rev. 2012;17(5):11–5.
5. Fukami T, Koide T, Hisada H, Inoue M, Yamamoto Y, Suzuki T, et al. Pharmaceutical evaluation of atorvastatin calcium tablets available on the Internet: A preliminary investigation of substandard medicines in Japan. J Drug Deliv Sci Technol. 2016;31:35–40.
6. Lanzarotta A, Lorenz L, Batson JS, Flurer C. Development and implementation of a pass/fail field-friendly method for detecting sildenafil in suspect pharmaceutical tablets using a handheld Raman spectrometer and silver colloids. J Pharm Biomed Anal [Internet]. 2017;146:420–5. Available from: http://www.elsevier.com/locate/jpba
7. Batson JS, Bempong DK, Lukulay PH, Ranieri N, Duane SR, Verbois L. Assessment of the effectiveness of the CD3+ tool to detect counterfeit and substandard anti-malarials. Malar J. 2016;15:119.
8. Degardin K, Guillemain A, Roggo Y. Comprehensive Study of a Handheld Raman Spectrometer for the Analysis of Counterfeits of Solid-Dosage Form Medicines. J Spectrosc. 2017;2017:1–13.
9. Ma B, Wang L. An application of rapid detection technologies in a national regulatory laboratory setting: Differentiating imported and domestic drug products of oxcarbazepine using handheld Raman, near infrared, and portable FTIR analyzers. Am Pharm Rev. 2015;18(2):no pagination.
10. Ma B, Huong LTT, Liu Y, Kamel MM, Zhao E. Rapid detection of counterfeit drugs of ethambutol hydrochloride and cefuroxime axetil using handheld raman, near infrared and portable FTIR technologies. Am Pharm Rev. 2014;17(5):54–61.
11. Kakio T, Yoshida N, Macha S, Moriguchi K, Hiroshima T, Ikeda Y, et al. Classification and Visualization of Physical and Chemical Properties of Falsified Medicines with Handheld Raman Spectroscopy and X-Ray Computed Tomography. Am J Trop Med Hyg. 2017;97(3):684–9.
12. Bate R, Tren R, Hess K, Mooney L, Porter K. Pilot study comparing technologies to test for substandard drugs in field settings. African J Pharm

Pharmacol. 2009;3(4):165–70.
13. Hajjou M, Qin Y, Bradby S, Bempong D, Lukulay P. Assessment of the performance of a handheld Raman device for potential use as a screening tool in evaluating medicines quality. J Pharm Biomed Anal. 2013;74:47–55.
14. Assi S, Watt R, Moffat T. Comparison of laboratory and handheld Raman instruments for the identification of counterfeit medicines. Spectrosc (Duluth, MN, United States). 2011;(Suppl.):36,38-44,46-47.
15. Bate R, Hess K. Anti-malarial drug quality in Lagos and Accra - A comparison of various quality assessments. Malar J. 2010;9(1).
16. Ricci C, Nyadong L, Yang F, Fernandez FM, Brown CD, Newton PN, et al. Assessment of hand-held Raman instrumentation for in situ screening for potentially counterfeit artesunate antimalarial tablets by FT-Raman spectroscopy and direct ionization mass spectrometry. Anal Chim Acta. 2008;623(2):178–86.
17. Sorak D, Herberholz L, Iwascek S, Altinpinar S, Pfeifer F, Siesler HW. New Developments and Applications of Handheld Raman, Mid-Infrared, and Near-Infrared Spectrometers. Appl Spectrosc Rev. 2012 Feb;47:83–115.
18. Le LMM, Tfayli A, Zhou J, Prognon P, Baillet-Guffroy A, Caudron E. Discrimination and quantification of two isomeric antineoplastic drugs by rapid and non-invasive analytical control using a handheld Raman spectrometer. Talanta. 2016;161:320–4.
19. Visser BJ, de Vries SG, Bache EB, Meerveld-Gerrits J, Kroon D, Boersma J, et al. The diagnostic accuracy of the hand-held Raman spectrometer for the identification of anti-malarial drugs. Malar J. 2016;15:160/1-160/12.
20. Assi S. Investigating the quality of medicines using handheld Raman spectroscopy. Eur Pharm Rev. 2014;19(5):56–60.
21. Corrigan DK, Salton NA, Preston C, Piletsky S. Towards the development of a rapid, portable, surface enhanced Raman spectroscopy based cleaning verification system for the drug nelarabine. J Pharm Pharmacol. 2010;62:1195–200.
22. United States Pharmacopoeial Convention. USP Technology Review: CBEx. 2017 [cited 2018 May 8]; Available from: http://www.usp.org/sites/default/files/usp/document/our-work/global-public-health/tr-report-cbex.pdf
23. Tondepu C, Toth R, Navin C V, Lawson LS, Rodriguez JD. Screening of unapproved drugs using portable Raman spectroscopy. Anal Chim Acta. 2017;973:75–81.
24. Alcala M, Blanco M, Moyano D, Broad NW, O'Brien N, Friedrich D, et al. Qualitative and quantitative pharmaceutical analysis with a novel hand-held miniature near infrared spectrometer. J Near Infrared Spectrosc. 2013;21(6):445–57.
25. Pederson CG, Friedrich DM, Hsiung C, von Gunten M, O'Brien NA, Ramaker H-J, et al. Pocket-size near-infrared spectrometer for narcotic materials identification. In: Next-Generation Spectroscopic Technologies VII, Proceedings of SPIE Vol 9101, 91010O. 2014. p. 91010O–1–11.
26. Wilson BK, Kaur H, Allan EL, Lozama A, Bell D. A New Handheld Device for the Detection of Falsified Medicines: Demonstration on Falsified Artemisinin-Based Therapies from the Field. Am J Trop Med Hyg. 2017 Feb;
27. Ishikawa D, Murayama K, Genkawa T, Awa K, Komiyama M, Ozaki Y. Development of a compact near infrared imaging device with high-speed and portability for pharmaceutical process monitoring. NIR news. 2012;23(8):14–7.
28. Ishikawa D, Murayama K, Awa K, Genkawa T, Komiyama M, Kazarian SG, et al. Application of a newly developed portable NIR imaging device to monitor the dissolution process of tablets. Vol. 405, Analytical and Bioanalytical Chemistry. Y. Ozaki; 2013. p. 9401–9.
29. Zontov Y V, Balyklova KS, Titova A V, Rodionova OY, Pomerantsev AL, Y.V. Z, et al. Chemometric aided NIR portable instrument for rapid assessment of medicine quality. J Pharm Biomed Anal. 2016;131:87–93.
30. Polli JE, Hoag SW, Flank S. Near-infrared spectrophotometric comparison of authentic and suspect pharmaceuticals. Pharm Technol. 2009;33(8):46–52.
31. Obeidat SM, Al-Tayyem BAN. Spectroscopic and chemometric analysis of illegally manufactured formulations of selected medicines. Orient J Chem. 2012;28(2):795–801.

32. Ranieri N, Tabernero P, Green MD, Verbois L, Herrington J, Sampson E, et al. Evaluation of a new handheld instrument for the detection of counterfeit artesunate by visual fluorescence comparison. Am J Trop Med Hyg. 2014;91(5):920–4.
33. Batson JS, Bempong DK, Lukulay PH, Ranieri N, Satzger RD, Verbois L. Assessment of the effectiveness of the CD3+ tool to detect counterfeit and substandard anti-malarials. Malar J. 2016;15(1):119.
34. Green MD, Hostetler DM, Nettey H, Swamidoss I, Ranieri N, Newton PN. Integration of novel low-cost colorimetric, laser photometric, and visual fluorescent techniques for rapid identification of falsified medicines in resource-poor areas: application to artemether-lumefantrine. Am J Trop Med Hyg. 2015;92(6 Suppl):8–16.
35. Lanzarotta A, Ranieri N, Albright D, Witkowski M, Batson J. Analysis Of counterfeit FDA-Regulated products at the forensic chemistry center: Rapid visual and chemical screening procedures inside and outside of the laboratory. Am Pharm Rev. 2015;18(3):no pagination.
36. Wilczyński S, Koprowski R, Błońska-Fajfrowska B. Directional reflectance analysis for identifying counterfeit drugs: Preliminary study. J Pharm Biomed Anal. 2016;124:341–6.
37. Bawuah P, Paakkonen P, Peiponen K-E. Gloss measurement in detection of surface quality of pharmaceutical tablets: a case study of screening of genuine and counterfeit antimalaria tablets. Acad-Rapid Publ. 2017;13:18.
38. Green MD, Nettey H, Villalva Rojas O, Pamanivong C, Khounsaknalath L, Grande Ortiz M, et al. Use of refractometry and colorimetry as field methods to rapidly assess antimalarial drug quality. J Pharm Biomed Anal. 2007 Jan;43(1):105–10.
39. Rodomonte AL, Gaudiano MC, Antoniella E, Lucente D, Crusco V, Bartolomei M, et al. Counterfeit drugs detection by measurement of tablets and secondary packaging colour. J Pharm Biomed Anal. 2010;53(2):215–20.
40. Guo S, Wang B, He L, Tisch DJ, Kazura J, Mharakurwa S, et al. Pilot testing of dipsticks as point-of-care assays for rapid diagnosis of poor-quality artemisinin drugs in endemic settings. Trop Med Health. 2016;44:15.
41. He L, Nan T, Cui Y, Guo S, Zhang W, Zhang R, et al. Development of a colloidal gold-based lateral flow dipstick immunoassay for rapid qualitative and semi-quantitative analysis of artesunate and dihydroartemisinin. Malar J. 2014;13:127/1-127/10, 10.
42. Guo S, Zhang W, He L, Tan G, Min M, Kyaw MP, et al. Rapid evaluation of artesunate quality with a specific monoclonal antibody-based lateral flow dipstick. Anal Bioanal Chem. 2016 Sep 12;408:6003–8.
43. Koesdjojo MT, Wu Y, Boonloed A, Dunfield EM, Remcho VT. Low-cost, high-speed identification of counterfeit antimalarial drugs on paper. Talanta. 2014;130:122–7.
44. Weaver AA, Reiser H, Barstis T, Benvenuti M, Ghosh D, Hunckler M, et al. Paper Analytical Devices for Fast Field Screening of Beta Lactam Antibiotics and Antituberculosis Pharmaceuticals. Anal Chem. 2013;85(13):6453–60.
45. Weaver AA, Lieberman M. Paper test cards for presumptive testing of very low quality antimalarial medications. Am J Trop Med Hyg. 2015;92(6 Suppl):17–23.
46. Myers N. Lab on a paper: adapting quantitative chemical techniques for use in low resource areas [Internet]. 2017 [cited 2018 May 9]. Available from: https://curate.nd.edu/downloads/und:m900ns08m33
47. Myers NM, Kernisan EN, Lieberman M. Lab on Paper: Iodometric Titration on a Printed Card. Anal Chem. 2015;87(7):3764–70.
48. Visser BJ, Meerveld-Gerrits J, Kroon D, Mougoula J, Vingerling R, Bache E, et al. Assessing the quality of anti-malarial drugs from Gabonese pharmacies using the MiniLab: A field study. Malar J. 2015;14(1):no pagination.
49. Risha PG, Msuya Z, Clark M, Johnson K, Ndomondo-Sigonda M, Layloff T. The use of Minilabs to improve the testing capacity of regulatory authorities in resource limited settings: Tanzanian experience. Health Policy. 2008;87(2):217–22.
50. Bate R, Jensen P, Hess K, Mooney L, Milligan J, R. B, et al. Substandard and falsified anti-tuberculosis drugs: a preliminary field analysis. Int J Tuberc Lung Dis. 2013;17(3):308–11.

51. Pribluda VS, Barojas A, Anez A, Lopez CG, Figueroa R, Herrera R, et al. Implementation of basic quality control tests for malaria medicines in Amazon Basin countries: results for the 2005-2010 period. Malar J. 2012;11:202.
52. World Health Organization. Survey of the quality of Selected antimalarial medicines circulating in Six countries of Sub-Saharan africa [Internet]. Geneva, Switzerland; 2011 [cited 2017 Aug 28]. Available from: http://apps.who.int/medicinedocs/en/d/Js17835en/
53. Risha P, Msuya Z, Ndomondo-Sigonda M, Layloff T. Proficiency testing as a tool to assess the performance of visual TLC quantitation estimates. J AOAC Int. 2006;89(5):1300–4.
54. Pan H, Ba-Thein W. Diagnostic Accuracy of Global Pharma Health Fund Minilab ™ in Assessing Pharmacopoeial Quality of Antimicrobials. Am J Trop Med Hyg. 2017;tpmd170289.
55. Desai D. PharmaChk: Robust device for counterfeit and substandard medicines screening in developing regions. [Internet]. 2014. Available from: https://open.bu.edu/handle/2144/12087
56. Bernier MC, Li F, Musselman B, Newton PN, Fernandez FM. Fingerprinting of falsified artemisinin combination therapies via direct analysis in real time coupled to a compact single quadrupole mass spectrometer. Anal Methods. 2016;8(36):6616–24.
57. Barras J, Katsura S, Sato-Akaba H, Itozaki H, Kyriakidou G, Rowe MD, et al. Variable-pitch rectangular cross-section radiofrequency coils for the nitrogen-14 nuclear quadrupole resonance investigation of sealed medicines packets. Anal Chem. 2012;84(21):8970–2.
58. Dunn JD, Gryniewicz-Ruzicka CM, Kauffman JF, Westenberger BJ, Buhse LF. Using a portable ion mobility spectrometer to screen dietary supplements for sibutramine. J Pharm Biomed Anal. 2011;54(3):469–74.
59. Gryniewicz CM, Reepmeyer JC, Kauffman JF, Buhse LF. Detection of undeclared erectile dysfunction drugs and analogues in dietary supplements by ion mobility spectrometry. J Pharm Biomed Anal. 2009;49:601–6.
60. Nguyen TAH, Pham TNM, Doan TT, Ta TT, Saiz J, Nguyen TQH, et al. Simple semi-automated portable capillary electrophoresis instrument with contactless conductivity detection for the determination of beta-agonists in pharmaceutical and pig-feed samples. J Chromatogr A [Internet]. 2014;1360:305–11. Available from: http://ovidsp.ovid.com/ovidweb.cgi?T=JS&PAGE=reference&D=emed16&NEWS=N&AN=604689706
61. United States Pharmacopoeial Convention. USP Technology Review: Speedy Breedy. [cited 2018 May 8]; Available from: http://www.usp.org/sites/default/files/usp/document/our-work/global-public-health/technology-review-report-speedy-breedy.pdf
